# Controlled Biocatalytic Synthesis of a Metal Nanoparticle‐Enzyme Hybrid: Demonstration for Catalytic H_2_‐driven NADH Recycling

**DOI:** 10.1002/anie.202404024

**Published:** 2024-05-28

**Authors:** Lucy B. F. Browne, Tim Sudmeier, Maya A. Landis, Christopher S. Allen, Kylie A. Vincent

**Affiliations:** ^1^ Department of Chemistry University of Oxford Inorganic Chemistry Laboratory South Parks Rd Oxford OX1 3QR United Kingdom; ^2^ Electron Physical Science Imaging Centre Diamond Light Source Oxford OX11 0DE United Kingdom; ^3^ Department of Materials University of Oxford Parks Rd Oxford OX1 3PH United Kingdom

**Keywords:** biocatalysis, biohybrid, chemoenzymatic, metal nanoparticle synthesis, nicotinamide cofactor regeneration

## Abstract

Here we demonstrate the preparation of enzyme‐metal biohybrids of NAD^+^ reductase with biocatalytically‐synthesised small gold nanoparticles (NPs, <10 nm) and core‐shell gold‐platinum NPs for tandem catalysis. Despite the variety of methods available for NP synthesis, there remains a need for more sustainable strategies which also give precise control over the shape and size of the metal NPs for applications in catalysis, biomedical devices, and electronics. We demonstrate facile biosynthesis of spherical, highly uniform, gold NPs under mild conditions using an isolated enzyme moiety, an NAD^+^ reductase, to reduce metal salts while oxidising a nicotinamide‐containing cofactor. By subsequently introducing platinum salts, we show that core‐shell Au@Pt NPs can then be formed. Catalytic function of these enzyme‐Au@Pt NP hybrids was demonstrated for H_2_‐driven NADH recycling to support enantioselective ketone reduction by an NADH‐dependent alcohol dehydrogenase.

## Graphical abstract

Under mild conditions, small (<10 nm), spherical, monodisperse metal nanoparticles (NPs) can be synthesised using an NAD^+^ reductase enzyme as reductant and stabilizer, forming Au NPs and Au@Pt (core‐shell) NPs. The enzyme‐Au@Pt NP hybrids demonstrated promising activity as a H_2_‐driven NADH cofactor recycling system, coupled with an alcohol dehydrogenase (ADH).

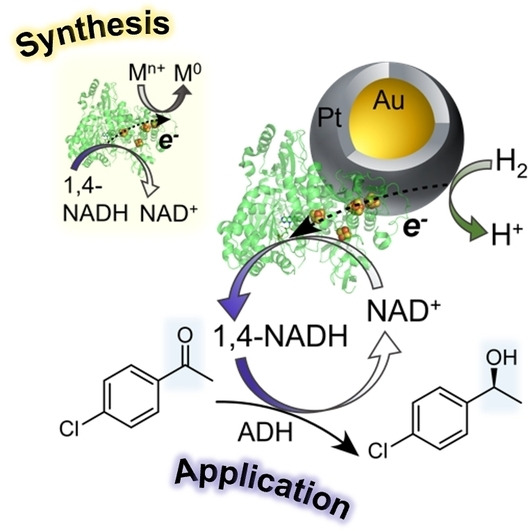

The need to maintain size and shape control over metal nanoparticles (NPs), while transitioning to more sustainable conditions for their synthesis and catalytic operation, present key drivers for finding new synthetic routes to NPs.[^1^, ^2^] Achieving high uniformity of metal NPs is crucial for applications ranging from catalysis, drug delivery and biosensing to electronics.^[3]^ In particular, gold NPs have received significant attention, largely due to their biocompatibility and optical properties.^[4]^ The traditional method for making gold NPs involves boiling tetrachloroauric acid (HAuCl_4_) and sodium citrate.[^5^, ^6^] This method produces larger sized NPs (10–200 nm, depending on the procedure) which have uses in biomedical applications. For catalysis, however, smaller sized NPs (<10 nm) are more desirable due to their increased surface area to volume ratio, with a higher number of catalytically active surface sites, leading to greater reactivity.^[7]^ Methods to make small Au NPs include using sodium borohydride as reductant with additional stabilising/capping agents such as alkane thiols or thiol‐containing polymers.[^8^, ^9^]

In search of more sustainable synthesis routes for gold NPs, numerous bio‐based strategies have been explored using plant extracts or micro‐organisms; these often allow milder conditions to be employed, however, the resulting NPs tend to have poor uniformity.[^10^, ^11^, ^12^] There have been some examples reported for using isolated enzymes in the synthesis of gold NPs, and these can give improved NP uniformity.[^13^, ^14^] These include utilising surface exposed thiols, on proteins, as reducing agents.^[13]^ Another example used a glutathione reductase enzyme where Au(III) reduction and size‐controlled NP formation occurred at the enzyme active site which however led to a loss of enzyme activity for its natural substrate.^[14]^

Although gold NPs show relatively low activity for many catalytic processes, gold is often used in combination with scarcer platinum group metals, either in a core‐shell or alloy, which can offer advantageous reactivity and/or stability properties.^[15]^ There are very few examples of synthesis of core‐shell NPs by “green” methods, and organic solvents which rate poorly on *GSK's Solvent Sustainability Guide*,^[16]^ such as toluene (rated amber), are common. Even syntheses involving aqueous conditions still require high temperatures or the use of metal surface‐binding ligands such as carbon monoxide to achieve uniform core‐shell structures.[^17^, ^18^, ^19^]

Here, we describe a pathway to synthesise metal NPs using an isolated enzyme which allows for mild synthesis conditions in water, while enabling high uniformity of NP size and shape. Specifically, we use an NAD^+^ reductase enzyme moiety (NRase), expressed in *E. coli* (HoxFU subunits of *Hydrogenophilus thermoluteolus* soluble hydrogenase, see Supporting Information, sections S. 1.3 and S. 2.1 for more details).^[20]^ NRase contains a bound flavin mononucleotide (FMN) which is the catalytic site for reversible oxidation of 1,4‐dihydronicotinamide adenine dinucleotide (hereafter, NADH), releasing electrons to the surface of the protein via a chain of iron‐sulfur (FeS) clusters. Here we exploit this directed flow of electrons during oxidation of NADH (or cheaper synthetic analogues) for reduction of metal salts to form biohybrid Au NPs, as well as Au core, Pt shell (Au@Pt) NPs, Scheme 1A.

**Scheme 1:**
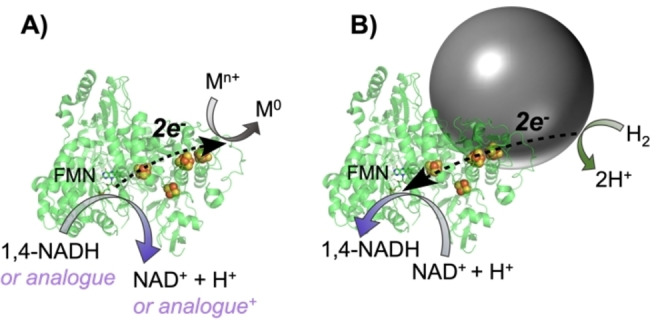
**A**) Synthesis of metal NPs using an NAD^+^ reductase moiety (NRase) and NADH or synthetic analogue as the reductant while metal salt (M^n+^) is reduced to M^0^. **B**) Depiction of H_2_ oxidation occurring at the metal NP surface and subsequent electron transfer to the NRase active site (FMN), where selective NAD^+^ reduction can occur.

We show that the biohybrid Au@Pt NPs have catalytic activity which differs from that of the metal NPs or NRase alone. Our results demonstrate that Au@Pt NPs made using the enzyme, NRase, can be used for their H_2_ oxidation properties, under mild pressure and temperature (e.g. 1 bar and 25 °C) to supply electrons back to NRase which selectively reduces NAD^+^ to the bioactive NADH cofactor (Scheme 1B). This provides a novel route for continuous, H_2_‐driven, atom‐efficient recycling of NADH. Use of H_2_ as reductant for NADH has been previously explored using methods including intact soluble hydrogenase enzymes;^[21]^ co‐immobilised hydrogenase and NRase on carbon particles;^[22]^ Pt nanoparticles;^[23]^ and homogeneous Ir catalysts.^[24]^ Despite having good selectivity, the Ir catalysts face bio‐incompatibility issues.^[25]^ A key limitation with Pt based systems is poor selectivity for making 1,4‐NADH, but in previous work we showed that this limitation could be overcome by adsorbing an NRase onto a commercial carbon‐supported platinum or palladium catalyst.[^26^, ^27^] In this work we investigate whether NPs made via a bio‐based method can obtain the desired selectivity for 1,4‐NADH; using the already‐present NRase and NPs together. As an example, we demonstrate coupling this with an NADH‐dependent alcohol dehydrogenase (ADH) for an enantioselective ketone reduction. NADH‐dependent enzymes are widely used in industrial biotechnology for fine chemical manufacturing, but are typically run with atom‐uneconomical, glucose‐driven, NADH recycling.^[28]^ Hence we demonstrate one application for the biohybrid Au@Pt NPs is in selective, H_2_‐driven NADH recycling to support the activity of ADH in an enantioselective ketone reduction.

First, to investigate the synthesis of metal NPs using NRase: NADH and HAuCl_4_ were added to different concentrations of NRase enzyme, Figure 1A. Over time the reaction solutions with NRase were observed to change colour, while the control, without enzyme, remained colourless (Figure 1A shows solutions after 16 h). As shown by the UV/Vis spectra, these colour changes correspond to the growth of a surface plasmon peak with the absorbance maximum (λ_max_) around 520 nm, indicative of Au NP formation, which was confirmed by TEM analysis (Figure 1B and inset). (The iron‐sulfur clusters of NRase absorb in the 400–500 nm region,^[20]^ and these spectral contributions increase with enzyme concentration; see Figure S3 for further spectra recorded at the start of the reaction.) The position and broadness of the surface plasmon peak for Au NPs is well‐established to show a correlation with NP size and uniformity.^[29]^ We use this correlation to estimate an average NP diameter for each reaction mixture (Figure 1C, triangle symbols), showing that NPs range from 5.4 nm (0.4 mg mL^−1^ NRase), down to 2.2 nm±0.1 nm (1.9 mg mL^−1^ NRase), with very small variation between experimental repeats (see S. 2.4 for more details). Thus, there is a strong correlation between the NP size and the concentration of NRase used (samples B to E).

**Figure 1:**
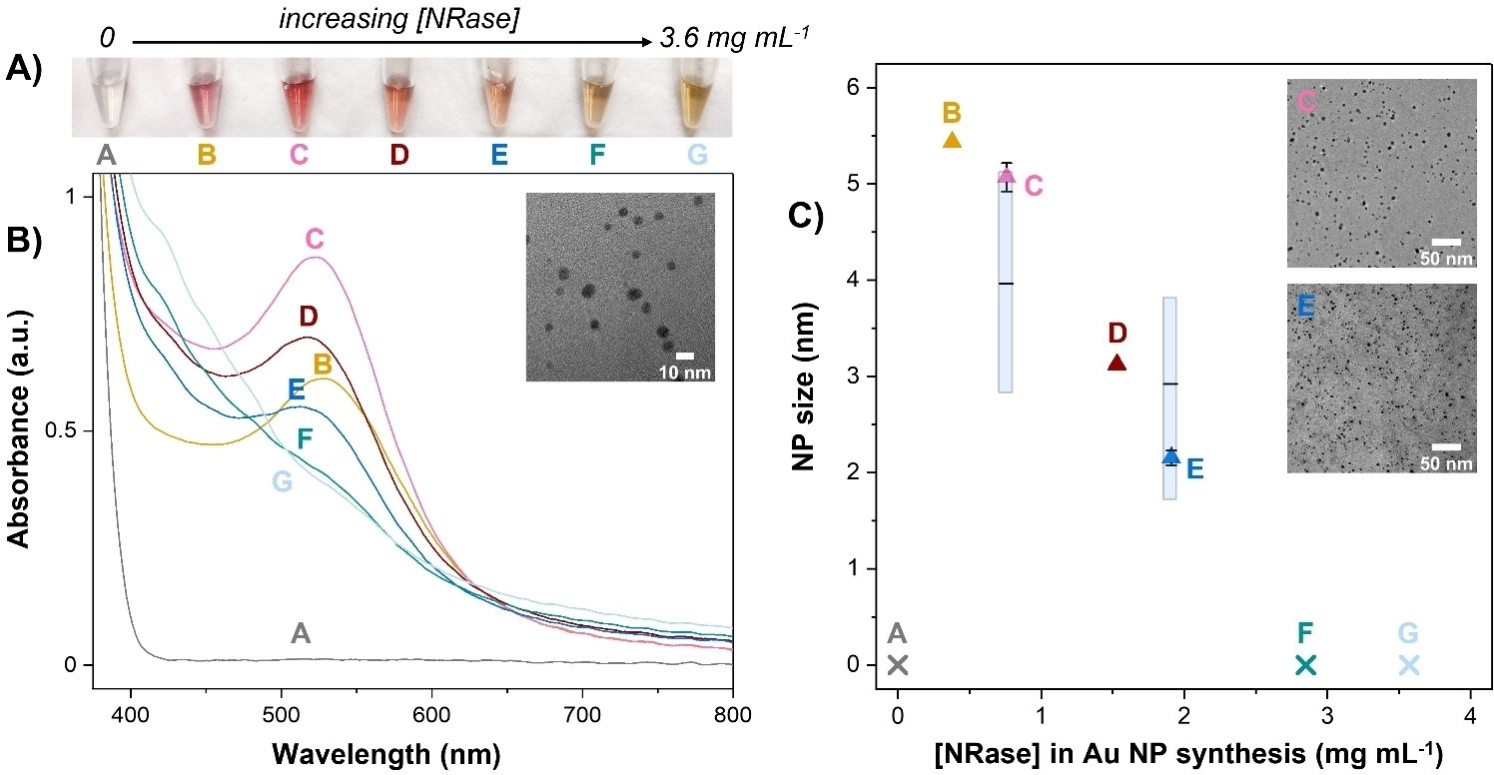
Size‐controlled synthesis of Au NPs using NRase and NADH. **A**) Photograph, taken after 16 h, of reactions all containing the same initial concentration of HAuCl_4_ and NADH, with different amounts of NRase (mg mL^−1^): A: 0, B: 0.4, C: 0.8, D: 1.5, E: 1.9, F: 2.9, G: 3.6. **B**) The corresponding UV/Vis spectra for the reactions photographed in A). The inset shows an example of a TEM image of these NPs (from sample C). **C**) The UV/Vis spectra can be used to estimate the average NP diameter, shown by the coloured symbols in this graph, with respect to the concentration of NRase used in the synthesis. The error bars are from results of replicate experiments. The vertical bars shown for C and E indicate the NP size distribution (capturing 25–75 % of the NP sizes, with the average shown by the black horizontal line) measured from TEM images such as those shown in the inset images (samples C and E pictured).

Nanoparticle formation and size distribution was corroborated from TEM analysis of samples C and E (see Figure 1C inset). TEM images showed spherical metal NPs with typical *fcc* lattice type with a size distribution in reasonable agreement with the UV/Vis analysis (vertical bars on Figure 1C; for details see S. 2.5 and S. 2.6). The trend of increasing NRase concentration leading to smaller NP sizes is consistent with particle seeding on the surface of the enzyme, where electrons are released via the FeS relay, since a higher concentration of enzyme leads to more nucleation sites and thus smaller particles (i.e. the same number of metal atoms are split between more sites). This is consistent with the NRase templating NP formation.[^30^, ^31^]

The NRase is highly selective for NADH over the related cofactor NADPH which differs only in an additional phosphate group (Figure S9) and has a very similar reduction potential.^[32]^ To confirm that NADH oxidation at the active site of NRase is critical for electron supply to allow NP synthesis, we ran experiments under identical conditions with NRase, Au(III) and either NADH or NADPH. The latter experiment remained colourless, and UV/Vis spectroscopy confirmed NP formation only in the experiment with NADH (Figure S10).

Given the high cost of the biological nicotinamide cofactors, we then explored whether the NP synthesis could be replicated with cheaper, synthetic cofactor analogues which are known to be tolerated by NRase.^[33]^ Two synthetic cofactors, 1‐benzyl‐1,4‐dihydronicotinamide (BNAH) and 1‐carbamoylmethyl‐1,4‐dihydronicotinamide (AmNAH),^[34]^ were found to give NPs with similar UV/Vis spectra, as when using NADH: all with a narrow surface plasmon peak with its maximum at the same wavelength (±1 nm) for a given NRase concentration (Figure S11A). Thus, this implies the size of the NPs from the synthesis in the presence of NRase is controlled by the enzyme and not by the type of cofactor/reductant used. Controls were also carried out without any NRase. In contrast to the control with NADH and no NRase, in which no NPs formed, the BNAH and AmNAH synthetic cofactors were able to cause reduction of Au(III) and formation of NPs. However, they gave poor size‐control over the NPs as evidenced by a very broad surface plasmon peak (Figure S11B) and subsequent precipitation, suggesting the cofactors could not provide long‐term stabilization of the NPs.

Experiments were then conducted to try to generate a Pt shell over the Au NP core by subsequent reduction of Pt(IV) by NRase using NADH as reductant. Figure 2A shows that the surface plasmon peak is dampened after the addition of Pt(IV) and further NADH to the preformed biohybrid NRase‐Au NPs. This is consistent with the formation of a core‐shell structure since the plasmon peak has been shown to decrease in absorbance when Au is no longer exposed.^[19]^ The average Au NP size in the starting NRase‐Au biohybrids was estimated to be 2.7±0.2 nm from the UV/Vis spectrum; after Pt(IV) reduction, TEM analysis showed that the average NP size was 6.4±2.4 nm (Figure S14). The Au−Pt NPs were also investigated by HR‐STEM‐EDX (Figure 2B and Figure S16). The EDX line scan data is consistent with a core‐shell structure, with a higher ratio of Pt versus Au detected in the outer layers of the NPs (0.9±0.4 nm Pt shell thickness) and a higher ratio of Au versus Pt in the centre (3.8±0.1 nm core). We subsequently refer to these as *biohybrid NRase‐Au@Pt NPs*.

**Figure 2:**
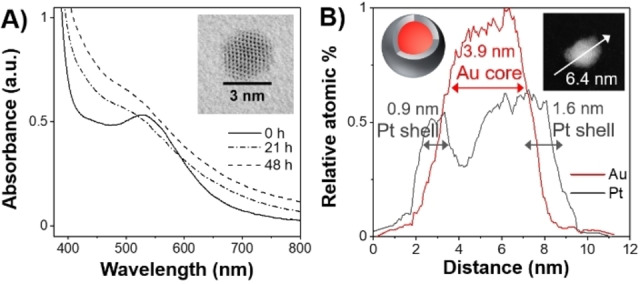
Synthesis of core‐shell Au@Pt NPs using NRase to reduce Au(III) followed by Pt(IV). **A**) UV/Vis spectra recorded after addition of K_2_PtCl_6_ and NADH to a solution of NRase and Au NPs (0 h) and after 21 and 48 h. Inset is a HR‐STEM (BF) image of a NP after 48 h. **B**) EDX data of the line scan shown in the inset HR‐STEM (HAADF) image, of sample after 48 h, showing a Pt shell and gold core, as depicted in the inset diagram (top left).

Two types of control experiments were performed. (i) “no NRase”: Pt(IV) was added to a reaction solution which had no NRase, but which had NADH and Au(III) “incubated” for the same length of time as the solutions used to form the NRase‐Au NPs. (ii) “1‐step”: Pt(IV) and Au(III) were added at the same time to a solution of NADH and NRase. Although these samples gave rise to a broad absorbance in the UV/Vis region (Figure S18), no evidence for defined core‐shell Au@Pt structures was observed in HR‐STEM‐EDX analysis. Instead, these reaction mixtures showed some evidence of Pt‐core Au‐layer and thin Pt‐shell, as well as alloy structures (Figure S17). These will be referred to as (i) *non‐bio‐Au‐Pt NPs* and (ii) *NRase‐Au‐Pt NPs*. Further control experiments under the same conditions, but without gold, showed only very low concentrations of Pt NPs in both the absence and presence of NRase (Figure S19). A catalytic role for gold in reduction of platinum salts has been shown previously in the case of making Au@Pt core‐shell nanostructures of around 50 nm in size using gallic acid, a mild reducing agent, where it was proposed that Au NPs can behave as seeds for Pt reduction.^[19]^ The Pt(IV) to Pt(II) and Pt(II) to Pt(0) couples have more negative reduction potentials than the Au(III) to Au(0) couple, hence Au(III) would be expected to be reduced before Pt(IV) in a mixture containing salts of both metals and a reducing agent.^[35]^

We then tested a series of NP samples for their selectivity and activity in NAD^+^ reduction using H_2_ as the electron source (as depicted in Scheme 1B). Using a non‐enzymatic catalyst for NAD^+^ reduction often leads to a mixture of products including 1,6‐NADH, 1,2‐NADH, NAD_2_ dimers as well as over‐reduced products (Figure S22), all of which cannot be utilised by enzymes such as alcohol dehydrogenase, which are selective for 1,4‐NADH (Figure S22).^[26]^ The *non‐bio‐Au‐Pt NPs* converted all NAD^+^, but after 21 hours, the ^1^H NMR spectrum showed no trace of 1,4‐NADH and instead showed exclusively over‐reduced products, i.e. with two double bonds on the pyridine ring now reduced (Table 1, entry 1 and Figure S24). The *biohybrid NRase‐Au@Pt NPs* prepared using different amounts of NRase were next compared (Table 1, entries 2–6).

**Table 1:** The selectivity and conversion in H_2_‐driven NAD^+^ reduction catalysed by *non‐bio‐Au‐Pt NPs* and *biohybrid NRase‐Au@Pt NPs* made using different amounts of NRase in the NP synthesis.

Entry	[NRase] (mg mL^−1^)^[a]^	Selectivity for 1,4‐NADH (%)^[b]^	Conversion of NAD^+^ (%)^[c]^
1	0	0	100
2	0.2	17	100
3	0.5	64	88
4^[d]^	0.8	74±<1	35±2
5	1.9	>99	20
6	3.2	n/a	0

[a] Concentration of NRase that was introduced during the synthesis of the NPs. [b] Selectivity measures the content of 1,4‐NADH as a percentage of the total reaction products, as analysed by ^1^H NMR spectroscopy. [c] Conversion measures the consumption of NAD^+^ starting material, as analysed by ^1^H NMR. [d] This entry reflects duplicate measurements (from two separate reaction set‐ups). Reaction conditions: NAD^+^ (1 mM) in potassium phosphate buffer (5 mM, pH 8.0), H_2_ (1 bar), 20–25 °C, 21 hours, using general procedure S. 1.7.1.

As the concentration of NRase used in the synthesis was increased, the selectivity for 1,4‐NADH increased, albeit with lower conversion. The decrease in conversion with increasing enzyme concentration can likely be explained by blocking of metal surface sites by additional enzyme. At an NRase concentration of 1.9 mg mL^−1^, solely 1,4‐NADH and NAD^+^ was observed, indicating that the enzyme is able to suppress any non‐selective reduction of NAD^+^ at the metal surface. We have previously observed a similar effect when using a biohybrid catalyst comprising NRase adsorbed onto a commercial carbon‐supported platinum or palladium catalyst, in which higher loadings of enzyme resulted in good selectivity for 1,4‐NADH (i.e. again suppressing the metal‐catalysed cofactor reduction).[^26^, ^27^] The fact that NRase remains catalytically active for NAD^+^ reduction in the biohybrid system provides strong evidence that the enzyme remains structurally intact and in electronic communication with the Pt where electrons are released from H_2_ oxidation. We therefore hypothesise that the metal NPs are formed on the enzyme surface, close to the external iron‐sulfur cluster, where electrons would be released from NADH oxidation during the NP synthesis for reduction of the metal salts, and where electrons would be taken up for NAD^+^ reduction during subsequent H_2_‐driven catalysis.

Lowering the Pt(IV) concentration used to form the Pt‐shell in *biohybrid NRase‐Au@Pt NPs* resulted in negligible conversion of NAD^+^ to NADH (Table S4, entries 7 and 8). Similarly, NPs made with Pt(IV) and Au(III) added at the same time, *NRase‐Au‐Pt NPs*, which were shown by HR‐STEM‐EDX to have a thinner outer Pt shell, gave no conversion (Figure S17, Table S4, entry 9). This suggests the need for significant coverage of the original Au NPs with Pt for H_2_ oxidation to drive NAD^+^ reduction. Overall, this illustrates that the structure of the metal NPs, as well as the presence of sufficient amounts of NRase in this biohybrid catalyst system are both crucial for achieving selective NAD^+^ reduction.

We further investigated this by adding a small amount of extra NRase (0.07 mg mL^−1^) to *biohybrid NRase‐Au@Pt NPs* and subjecting to NAD^+^ reduction conditions. The presence of NRase added after the NP synthesis was found to significantly increase conversion of NAD^+^ as well as selectivity for 1,4‐NADH (Table S4, compare entries 3 and 4 with entries 12 and 13 respectively). For example, improving the result obtained with *biohybrid NRase‐Au@Pt NPs* made using 0.5 mg mL^−1^ NRase from 88 % conversion, with 64 % selectivity, to >99 % conversion to exclusively 1,4‐NADH. Adding this same small amount of NRase to *non‐bio‐Au‐Pt NPs* dramatically improved the selectivity from 0 % to 96 % 1,4‐NADH (with 4 % over‐reduced product) with both conditions showing no remaining starting material (Table S4, compare entries 1 and 11). These experiments clearly demonstrate the effect of NRase in providing the required selectivity. To explore the stability of the *biohybrid NRase‐Au@Pt NPs* under reaction conditions, the catalyst system was put through 3 cycles of re‐use (Table S4, entries 12, 14 and 15). Although conversion dropped, likely in part due to difficulty in recovering the catalyst system at the small scale of these reactions, importantly, there was no loss in selectivity for 1,4‐NADH, indicating that the NRase remains stable and in effective electronic communication with metal NPs.

Finally, the NPs with NRase were tested as an atom‐efficient 1,4‐NADH recycling catalyst for the enantioselective ketone reduction of 4′‐chloroacetophenone using an alcohol dehydrogenase (Scheme 2). From a practical point, it is noteworthy to mention that the biohybrid NPs were made in batches, flash frozen in liquid N_2_, and stored at −80 °C, before being slowly thawed (as per standard long‐term storage procedures for handling enzymes) before use in these reactions. No aggregation or change in the UV/Vis spectra was observed after thawing (Figure S31). High conversions, e.g. 84 %, of the (*S*)‐alcohol product were obtained, from analysing the reaction after 21 hours (higher conversions were achieved with longer reaction times, see Table S5 for more results). Importantly, no dechlorination was detected (by ^1^H NMR or GC analysis) and chiral‐GC analysis confirmed the (*S*)‐enantiomer was formed selectively (≥99 % e.e.). In comparison, commercial platinum or palladium on carbon catalysts often cause dehalogenation of organic substrates under hydrogenation conditions.^[26]^ Thus, this demonstrates the advantage enzymatic biohybrid reactions can provide, over traditional chemical methods, in enabling the use of mild conditions—e.g. 25 °C and 1 bar H_2_ pressure—to allow greater chemo‐ and enantio‐selectivity.

**Scheme 2:**
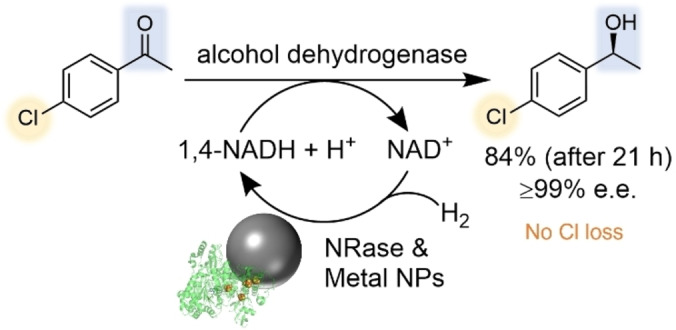
Enantioselective ketone reduction of 4′‐chloroacetophenone using gold‐platinum NPs with NRase as a H_2_‐driven NADH recycling catalyst.

In summary we have demonstrated how an isolated enzyme can be employed to synthesise Au NPs with relative ease, under mild conditions, and yielding high uniformity of NP size and shape, which can be further modified with a Pt shell. The reactivity of the NRase enzyme used to synthesise the metal NPs is retained in the final enzyme‐metal NP hybrids, such that the composite catalysts exhibit properties of both the enzyme and metal. This is demonstrated in selective, H_2_‐driven recycling of 1,4‐NADH, which is further coupled to an alcohol dehydrogenase, for enantioselective ketone reduction. This approach provides a blueprint for a new type of self‐synthesised chemo‐biocatalyst system which is likely to find wide‐ranging applications in areas of biotechnology.

## Conflict of interests

The authors declare no conflict of interest.

## Supporting information

As a service to our authors and readers, this journal provides supporting information supplied by the authors. Such materials are peer reviewed and may be re‐organized for online delivery, but are not copy‐edited or typeset. Technical support issues arising from supporting information (other than missing files) should be addressed to the authors.

Supporting Information

## Data Availability

The data that support the findings of this study are available in the supplementary material of this article.
