# Supplementary material for: Controlled Biocatalytic Synthesis of a Metal Nanoparticle‐Enzyme Hybrid: Demonstration for Catalytic H2‐driven NADH Recycling
Source: Angew Chem Int Ed Engl. 2024 May 28;63(27):e202404024. doi: 10.1002/anie.202404024 (PMC11497223; doi:10.1002/anie.202404024)
Supplement: Supplementary file 1 — Supporting Information [file ANIE-63-e202404024-s001.pdf]

Angewandte Chemie
Eine Zeitschrift der Gesellschaft Deutscher Chemiker

Supporting Information

Controlled Biocatalytic Synthesis of a Metal Nanoparticle-Enzyme Hybrid: Demonstration for Catalytic H_2-driven NADH Recycling

*L. B. F. Browne, T. Sudmeier, M. A. Landis, C. S. Allen, K. A. Vincent**

Supporting Information
©Wiley-VCH 2021
69451 Weinheim, Germany

Controlled Biocatalytic Synthesis of a Metal Nanoparticle-Enzyme Hybrid: Demonstration for Catalytic H_2-driven NADH Recycling

Lucy B. F. Browne, Tim Sudmeier, Maya A. Landis, Christopher S. Allen, and Kylie A. Vincent*

Abstract: Here we demonstrate the preparation of enzyme-metal biohybrids of NAD^+ reductase with biocatalytically-synthesised small gold nanoparticles (NPs, <10 nm) and core-shell gold-platinum NPs for tandem catalysis. Despite the variety of methods available for NP synthesis, there remains a need for more sustainable strategies which also give precise control over the shape and size of the metal NPs for applications in catalysis, biomedical devices, and electronics. We demonstrate facile biosynthesis of spherical, highly uniform, gold NPs under mild conditions using an isolated enzyme moiety, an NAD^+ reductase, to reduce metal salts while oxidising a nicotinamide-containing cofactor. By subsequently introducing platinum salts, we show that core-shell Au@Pt NPs can then be formed. Catalytic function of these enzyme-Au@Pt NP hybrids was demonstrated for H_2-driven NADH recycling to support enantioselective ketone reduction by an NADH-dependent alcohol dehydrogenase.

DOI: 10.1002/anie.2021XXXXX

Table of Contents

S.1 Experimental Procedures ... 3
S.1.1 General Reagents ... 3
S.1.2 General Method Considerations ... 3
S.1.3 Enzymes ... 3
S.1.4 Gold NP Preparation using NRase ... 3
S.1.5 Gold-Platinum NP Preparation ... 3
S.1.6 Characterisation of NPs ... 4
S.1.6.1 UV-visible Spectroscopy ... 4
S.1.6.2 TEM Imaging ... 4
S.1.6.3 HR-STEM with EDX Analysis ... 4
S.1.7 Reaction Methods ... 4
S.1.7.1 Gold-Platinum NPs Catalysed NAD^+ Reduction ... 4
S.1.7.2 4'-Chloroacetophenone Reduction using NADH Recycling ... 4
S.1.8 Reaction Analysis ... 5
S.1.8.1 ^1H NMR Spectroscopy ... 5
S.1.8.2 UV-visible Spectroscopy ... 5
S.1.8.3 Chiral-GC Method ... 5
S.2 Results and Discussion ... 6
S.2.1 Enzyme Details ... 6
S.2.2 Redox Potentials ... 7
S.2.3 Gold NP Synthesis UV-vis Spectra ... 7
S.2.4 Gold NP Size Calculation ... 8
S.2.5 Gold NP Size Distribution from TEM ... 9
S.2.6 Gold NP Lattice Fringe Analysis ... 10
S.2.7 Cofactor Studies ... 12
S.2.8 Gold-Platinum NPs Size Distribution from TEM ... 14
S.2.9 Gold-Platinum NPs HR-STEM-EDX Analysis ... 15
S.2.10 1-Step Versus 2-Steps for Gold-Platinum NP Formation ... 17
S.2.11 Control without Gold for Platinum NPs ... 18
S.2.12 Comparison of Using Different Ratios of Pt(IV) to Au(III) ... 19
S.2.13 Monitoring the Rate of Au NP Formation ... 19
S.2.14 NAD^+ Reduction Studies ... 20
S.2.15 4'-Chloroacetophenone Reduction ... 23
S.2.16 4'-Chloroacetophenone Reduction Analysis ... 25
S.2.17 Stability of NPs ... 28
S.3 References ... 28
S.4 Author Contributions ... 28

S.1 Experimental Procedures

S.1.1 General Reagents

All commercial reagents were used as received, without any further purification. NAD^+ and 1,4-NADH were purchased from Prozomix. Synthetic cofactors: 1-benzyl-1,4-dihydronicotinamide (BNAH) and 1-carbamoylmethyl-1,4-dihydronicotinamide (AmNAH) were received from Caroline Paul, Delft University of Technology. 4'-Chloroacetophenone was purchased from Alfa Aesar. Remaining reagents were purchased from Sigma-Aldrich. All aqueous solutions were prepared with deionized Milli-Q water (Millipore, ≥15 MΩcm).

S.1.2 General Method Considerations

To reduce any competing oxidation reactions, all reaction handling (including NP preparation procedures) was carried out in a glovebox, filled with a N_2 atmosphere. When handling metal salts including while preparing the NPs, care was taken to protect from exposure to light by covering in tinfoil (to eliminate any light-induced metal reduction). For “washing steps” used for removing small molecules from enzyme or NP solutions, 10 kDa Amicon ultra-0.5 centrifugal filter units (approximately 1 nm pore size, stated on Sigma-Aldrich website), purchased from Sigma-Aldrich were used. These were always washed with water prior to first use (by centrifuging at 12,000 rpm for 5 mins, x5) as recommended to remove residual glycerol found in the filters.

S.1.3 Enzymes

The NAD^+ reductase enzyme moiety, which we have abbreviated to “NRase”, is the NAD^+ reducing/NADH oxidizing subunit (also known as HoxFU where HoxF: 66 kDa and HoxU: 26 kDa, PDB code: 5XF9) of a soluble hydrogenase from *Hydrogenophilus thermoluteolus* without the hydrogenase subunit. NRase was expressed heterologously in *E. coli.*, isolated and purified using standard protocols. Before use in NP preparation and NADH recycling reactions, NRase (from stock flash frozen at -80 °C and stored in 50 mM Tris-HCl pH 8 buffer) was washed with pH 8 water (NaOH added to adjust pH) using 10 kDa size exclusion filter to remove storage buffer salts (by centrifuging at 12,000 rpm for 10 mins, x3). The alcohol dehydrogenase (ADH-105) used for the ketone reduction was received in lypophilised form from Johnson Matthey, Cambridge. Stock solutions of ADH-105 were prepared in 5 mM potassium phosphate pH 8 buffer and washed using a 10 kDa size exclusion filter to remove any undesired smaller proteins/reductant molecules that may be present (by centrifuging at 12,000 rpm for 10 mins, x3). ADH-105 has previously been shown to be selective for 1,4-NADH cofactor and selective in the reduction of acetophenone for the (*S*)-enantiomer of 1-phenylethanol.[1]

S.1.4 Gold NP Preparation using NRase

Typical small-scale screening experiments were carried out by adding the following reagents, in order, to 1.5 mL plastic tubes (StarLabs): NRase (e.g. from a stock of 7.6 mg mL^{-1} in pH 8 water), pH 8 water (adjusting the amount of NRase and pH 8 water to obtain the desired NRase concentrations) followed by 1,4-NADH (1 mM final concentration in pH 8 water) and finally $HAuCl_4$ (from an aqueous stock solution with NaOH added to adjust to pH 8, 0.7 mM final concentration). Thus a 1: 1.4 molar ratio of Au^{3+}: NADH was used. The reaction solutions were left at ambient temperatures without any stirring/shaking. After the desired time (e.g. 16 h), the NPs were washed using a 10 kDa size exclusion filter (by centrifuging at 12,000 rpm for 10 mins, until the UV-vis of the filtrate indicated minimal remaining NADH/NAD^+ and gold salts). These could then be used directly in the NAD^+ reduction experiments or in combination with ADH for an enantioselective ketone reduction.

S.1.5 Gold-Platinum NP Preparation

A typical procedure followed a 2-step approach where gold NPs were first prepared using NRase (as described in **S.1.4**) then before any washing steps, more 1,4-NADH was added (0.8 mM in pH 8 water) and K_2PtCl_6 (0.38 mM final concentration in pH 8 water). Thus, using a 1: 2.1 molar ratio of Pt^{4+}: NADH for this step and a 1: 1.1 molar ratio of Au^{3+}: Pt^{4+}. Typically, this solution was left for 48 hours after the addition of NADH and K_2PtCl_6 in order to allow for maximum Pt reduction. After which the solution can be washed using the same procedure described in **S.1.4** to remove any excess metal salts and NADH/NAD^+.

S.1.6 Characterisation of NPs

UV-visible spectroscopy was used to monitor the NP formation *in situ* or at time points throughout the reaction. Transmission electron microscopy (TEM) imaging and analysis was then carried out on samples after the NPs had been washed to remove any residual reactants, to give information on particle shape and size distribution. Selected gold-platinum NP samples were also analysed by high-resolution scanning transmission electron microscopy (HR-STEM) with energy-dispersive x-ray (EDX) spectroscopy to give information on elemental composition of the particles.

S.1.6.1 UV-visible Spectroscopy

UV-visible spectroscopy was carried out using a Cary 60 UV-visible spectrophotometer (Agilent) using a plastic micro-cuvette (path length 1 cm). For monitoring reaction progress of NP formation, the reaction solution was added to the micro-cuvette and the spectra was recorded from 200 to 800 nm. The increase in absorbance across the 400-800 nm range indicated the presence of scattering caused by NPs. For gold NPs a surface plasmon resonance (SPR) peak with an absorbance maximum in the 500-550 nm range was clear indication that gold NPs were formed. The ratio of the absorbance at the SPR peak to the absorbance at 450 nm was used to estimate the average NP size.[2] To observe the NADH oxidation to NAD^+, small aliquots of the NP reaction solutions were diluted with pH 8 water by a factor of 10 (e.g. 10 µL reaction solution and 90 µL pH 8 water).

S.1.6.2 TEM Imaging

Dilute samples were dropcast onto copper grids (type: holey carbon 300 mesh Cu TEM grids, S147-3 from Agar Scientific) and dried under vacuum. TEM was performed using a JEOL-3000F microscope operated at 200 kV.

S.1.6.3 HR-STEM with EDX Analysis

Dilute samples were dropcast onto copper grids (type: holey carbon 300 mesh Cu TEM grids, S147-3 from Agar Scientific) and dried under vacuum. HR-STEM was performed using a JEOL ARM300F microscope operated at 300 kV and this was coupled to an EDX detector (Oxford Instruments XMAX 100 EDX detector). For further elemental mapping studies, HR-STEM was performed using a JEOL ARM200F microscope operated at 200 kV, coupled to EDX and EELS detectors (JEOL Centurio EDX detector and Gatan Quantum Dual EELS spectrometer).

S.1.7 Reaction Methods

The following reaction methods were used for investigating the selectivity and activity of gold-platinum NPs prepared using NRase (and compared to those prepared without NRase) for the application as H_2-driven NADH recycling catalysts.

S.1.7.1 Gold-Platinum NPs Catalysed NAD^+ Reduction

A typical small-scale procedure used a 550 µL total reaction volume inside a 600 µL sized plastic tube (StarLabs). A stock solution of 1.9 mM NAD^+ in 5 mM potassium phosphate pH 8 buffer was prepared. Then gold-platinum NP solutions (e.g. 160 µL) were added to plastic tubes followed by 90 µL of 5 mM potassium phosphate pH 8 buffer (or if adding extra NRase, then the required amount of NRase and the remaining volume of 5 mM potassium phosphate pH 8 buffer to make up to 90 µL). Then 300 µL of the 1.9 mM NAD^+ solution was added to each plastic tube, making a 1 mM concentration of NAD^+ in the reaction solutions. The lids of each plastic tube were pierced with a needle and placed inside a Büchi Tinyclave pressure vessel which was then filled with H_2 (to 2.5 bar and vented x3 and then filled to 1 bar). The pressure vessel was then placed on a Stuart® mini see-saw rocker set to 30 oscillations/min. For analysis of the reaction products, the procedure described in **S.1.8** was followed.

S.1.7.2 4'-Chloroacetophenone Reduction using NADH Recycling

A typical small-scale procedure used a 550 µL total reaction volume inside a 600 µL sized plastic tube (StarLabs). A stock solution of 1.9 mM NAD^+ in 5 mM potassium phosphate pH 8 buffer was prepared. Then gold-platinum NP solutions (e.g. 160 µL) were added to plastic tubes followed by NRase and 5 mM potassium phosphate pH 8 buffer (making up their combined volume to 74.5 µL). Then 300 µL of the 1.9 mM NAD^+ solution was added to each plastic tube, making a 1 mM concentration of NAD^+ in the reaction solutions. Then 10 µL of an ADH-105 stock solution (24 mg mg mL^{-1} in 5 mM potassium phosphate pH 8 buffer) was added (making a 0.44 mg mL^{-1} ADH-105 concentration). Lastly 5.5 µL of a 0.5 M stock solution of was added (for example as a solution in acetonitrile) to make a 5

mM 4'-chloroacetophenone concentration with 1 vol% of the co-solvent in the reaction solution. The lids of each plastic tube were pierced with a needle and placed inside a Büchi Tinyclave pressure vessel which was then filled with H_2 (to 2.5 bar and vented x3 and then filled to 1 bar). The pressure vessel was then placed on a Stuart® mini see-saw rocker set to 30 oscillations/min. For analysis of the reaction products, the procedure described in **S.1.8** was followed.

S.1.8 Reaction Analysis

To confirm and quantify reaction products, ^1H NMR spectroscopy was used as the primary analytical technique. UV-visible spectroscopy was used to obtain more analysis from different time points across a NAD^+ reduction reaction, but ^1H NMR was required to confirm which NADH isomers were formed. A chiral-GC method was used for the confirmation of the enantioselectivity of the alcohol product from the 4'-chloroacetophenone reduction.

S.1.8.1 ^1H NMR Spectroscopy

After the desired reaction time, the reaction solution was added to a 10 kDa size exclusion filter and centrifuged for a total of 20 minutes at 12,000 rpm. The filtrate was then used for analysis and the residue in the filter (containing NPs and enzyme) could be re-used in a second reaction if desired. A total volume of 500 µL solution was prepared containing 400 µL of the filtered reaction solution and 100 µL of D_2O in a Norell® SelectSeries™ 5 mm 400 MHz NMR spectroscopy tube. All NMR spectra were obtained using a Bruker Advance III HD nanobay (400 MHz) instrument, a water suppression was applied and parameters used for acquiring NMR spectra are summarized in **Table S 1**. Data was processed using MestReNova. Manual rephasing was carried out when required followed by a Whittaker smoother baseline correction across the whole spectrum and a line broadening to improve signal to noise corresponding to 1.0 Hz. Signals were referenced against appropriate peaks such as acetonitrile (δ = 2.06 ppm).

Table S 1. NMR parameters used to acquire NAD^+ reduction and 4'-chloroacetophenone reduction product composition.

Experiment	^1H
RF pulse energy (MHz)	400.13
Temperature (K)	298
Number of scans	As required
Pulse width (µs)	14
Spectral width (Hz)	8000
Acquisition time (s)	4.09
Relaxation delay (s)	1.00

S.1.8.2 UV-visible Spectroscopy

UV-visible spectroscopy was carried out using a Cary 60 UV-visible spectrophotometer (Agilent) using a plastic micro-cuvette (path length 1 cm). To observe the NAD^+ reduction to NADH, small aliquots of the reaction solutions were diluted to make the initial NAD^+ concentration 0.1 mM. The increase in absorbance at 340 nm can be used as indication of the formation of NADH products. 1,4-NADH has absorbance maxima at 340 nm, however 1,6-NADH and 1,4-NADH also absorb at that wavelength (with their maxima at 345 nm and 395 nm respectively).[3] The observation of another peak around 290 nm indicated over-reduced NADH which was identified by ^1H NMR analysis.

S.1.8.3 Chiral-GC Method

After the desired reaction time, the reaction solution was added to a 10 kDa size exclusion filter and centrifuged for a total of 20 minutes at 12,000 rpm. The filtrate was then used for analysis. 50 µL of the filtrate was added to 800 µL EtOAc to extract the organic products. 600 µL of the EtOAc layer was removed, dried using Na_2SO_4, and added to a GC vial. The GC method was as described in **Table S 2**.

Table S 2. GC method conditions used for the enantiomeric excess (e.e.) determination of the reaction products from the reduction of 4'-chloroacetophenone.

Column	CP-Chirasil-Dex CB (Agilent), 25 m x 0.25 mm x 0.25 µm, fitted with a guard of 10 m undeactivated fused silica of the same diameter
Carrier	He (CP grade), 170 kPa (constant pressure)
Inlet temperature	200 °C
Injection conditions	Splitless with split flow 60 mL/min, splitless time 0.8 mins, purge 5 mL/min. Injection volume = 0.1 µL.
Detection	FID (H_2 = 35 mL/min, air = 350 mL/min, makeup N_2 = 40 mL/min, temp = 250 ℃)
Oven heating profile	Time (minutes) Temperature<br>0 – 5 Hold at 70℃<br>5 – 14.5 Ramp to 165 ℃ at 10 ℃/min<br>14.5 – 15.2 Ramp to 200 ℃ at 50 ℃/min<br>15.2 – 20.2 Hold at 180 ℃ for 5 minutes
Compound retention times	Time (minutes) Compound<br>12.99 4'-Chloroacetophenone.<br>14.95 *(R)*-4'-Chloro-1-phenylethanol<br>15.10 (*S*)-4'-Chloro-1-phenylethanol

S.2 Results and Discussion

S.2.1 Enzyme Details

The NAD^+ reductase enzyme moiety (NRase) used in this work consists of the HoxFU subunits from *Hydrogenophilus thermoluteolus* soluble hydrogenase. The x-ray crystal structure of the whole soluble hydrogenase (PDB code: 5XF9)[4] determines there to be 4 unique protein chains: HoxF, HoxU, HoxY and HoxH (**Figure S 1**). The NRase subcomplex performs NAD^+ reduction/NADH oxidation while the HoxHY functions as a hydrogenase: active for H^+ reduction and H_2 oxidation. The hydrogenase moiety in intact soluble hydrogenases is found to be unstable over time,[5] while the NAD^+ reductase moiety has good stability. Thus, in this work we have prepared and isolated the NRase subcomplex and coupled it with a metal NP in place of the HoxHY subcomplex.

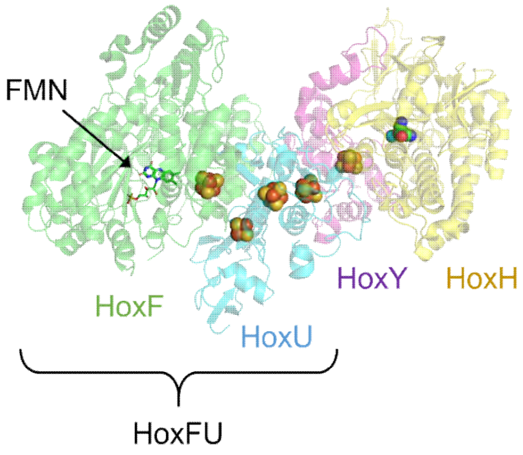

Figure S 1. Structure of NAD^+ reducing soluble hydrogenase (PDB: 5XF9) where the HoxFU subunit is what we have termed NRase in this work.

During NAD^+ reduction, 2 e^- need to be supplied to the FMN in the active site of NAD^+ reductase, via the FeS clusters. In the whole soluble hydrogenase these electrons will have originated from the hydrogenase subunit carrying out H_2 oxidation. In this work we have coupled a metal NP (specifically Au@Pt NPs), which carries out the H_2 oxidation, with the NRase subunit. In the NRase subunit, the configuration of amino acids surrounding the FMN in the active site only allows for the NAD^+/NADH to align itself in one configuration with respect to the FMN and thus causes the selectivity for the hydride transfer to occur at the 4-position of the pyridine ring. Therefore NAD^+ is reduced selectively to 1,4-NADH in the presence of the NAD^+ reductase enzyme (**Figure S 2**). The hydride will also only be transferred onto the *si* face of the NAD^+ therefore the reaction is also stereoselective which can be utilized in the presence of D_2O where the resulting product will be exclusively [4*S*-^{2}H]-NADH.[6] The whole reaction is reversible (the direction of the arrows drawn in **Figure S 2** can all be reversed). Thus, if no electrons are being supplied to the active site and there is 1,4-NADH present, NRase can oxidize NADH (by the hydride transferring onto FMN) and subsequently electrons are directed away from the active site, via the FeS clusters, to the surface of NRase.

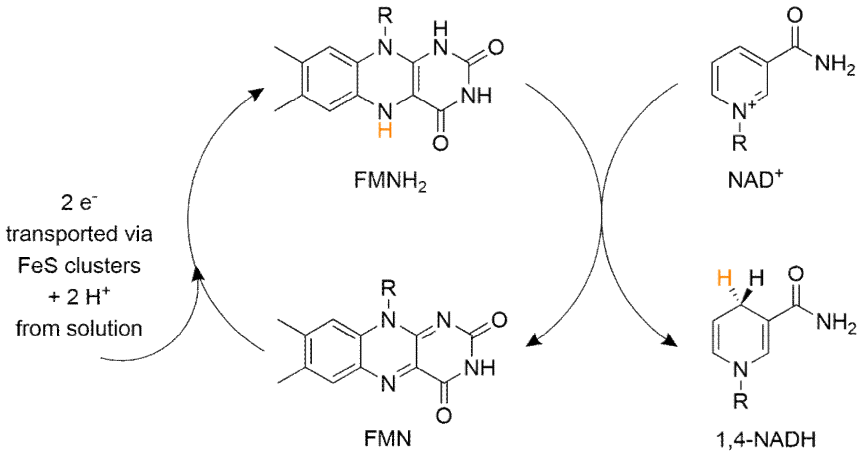

Figure S 2. Mechanism for NAD^+ reduction via hydride transfer from reduced FMN in active site of NRase. Arrows can be reversed for NADH oxidation, resulting in electrons directed towards surface of the NRase.

S.2.2 Redox Potentials

These redox potentials show that the oxidized cofactors have more negative reduction potentials than the metals, thus the reduced form of these cofactors can be used as sacrificial reductants to reduce Au(III) or Pt(IV).

Half-reaction	E°(vs SHE)/ V	Reference
$[AuCl_4]^- + 3e^- \rightleftharpoons Au_{(s)} + 4Cl^-$	1.00, (0.77 at pH 8)[a]	[7], [8]
$PtCl_6^{2-} + 2e^- \rightleftharpoons PtCl_4^{2-} + 2Cl^-$	0.726	[9]
$PtCl_4^{2-} + 2e^- \rightleftharpoons Pt_{(s)} + 4Cl^-$	0.758	[9]
$NAD^+ + 2e^- + H^+ \rightleftharpoons NADH$	-0.320	[10]
$NADP^+ + 2e^- + H^+ \rightleftharpoons NADPH$	-0.320	[10]
$BNA^+ + 2e^- + H^+ \rightleftharpoons BNAH$	-0.361	[11]
$AmNA^+ + 2e^- + H^+ \rightleftharpoons AmNAH$	-0.385	[11]

[a] This reference has measured a reduction potential of an aqueous solution of $HAuCl_4$ prepared at pH 8 of 0.53 V using a saturated calomel electrode (SCE). Solutions prepared at pH 6.2 and 2.9 were found to give reduction potentials of 0.59 V and 0.66 V respectively. At higher pH the speciation of the Au complexes are known to have a lower x number in $[AuCl_x(OH)_{4-x}]^-$ which gives rise to the lower reduction potentials.

S.2.3 Gold NP Synthesis UV-vis Spectra

The synthesis of gold NPs was tested with increasing amounts of NRase, as was discussed in the main text. **Figure S 3** shows the UV-vis spectra of all results from this experiment (including replicates which were omitted from Figure 1 for clarity). The replicates give very similar spectra after 16 h showing good reproducibility.

The trend of the blue-shift in the wavelength of the maxima of the surface plasmon resonance peak (λ_{max}) as the concentration of NRase is increased has been described in the main text. The other noticeable feature is there is an increase in the absorbance at the λ_{max} (from 0 to 0.8 mg mL^{-1} of NRase) followed by a decrease in absorbance at higher NRase concentrations. The initial increase in absorbance is matched with a decrease in the λ_{max} which implies that the concentration of NPs increases. Thereafter, the decrease in

absorbance cannot be assumed to be solely due to a decreasing NP concentration since the size of the NPs decreases and smaller NPs are known to have a smaller extinction coefficient.[12]

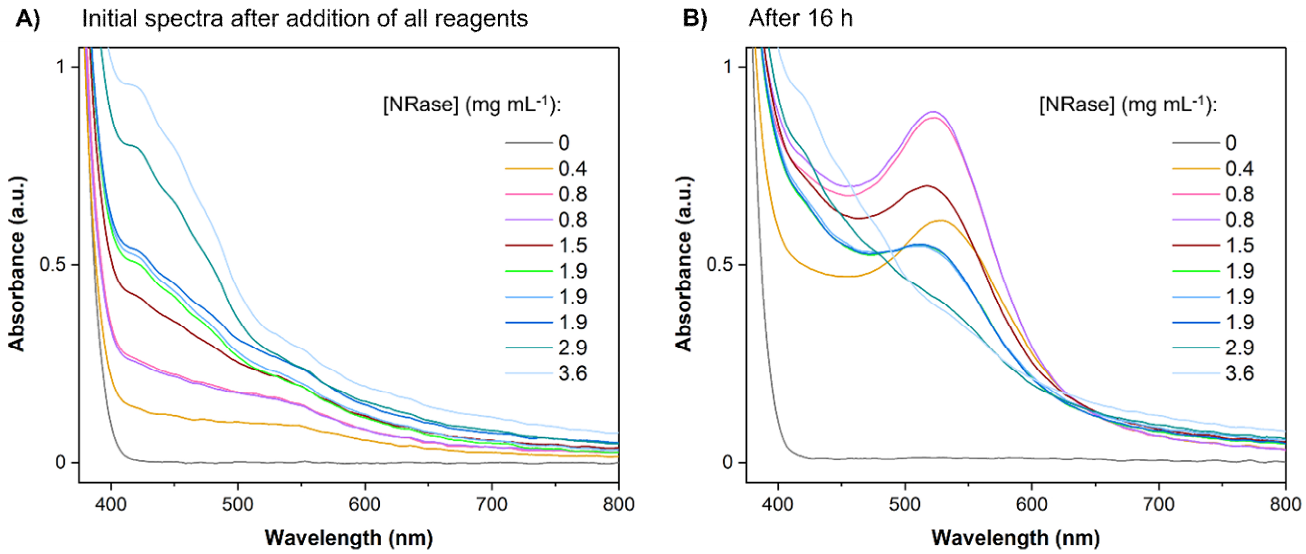

Figure S 3. Monitoring the synthesis of Au NPs using UV-vis. **A)** Spectra recorded after addition (within first 20 minutes) of all reagents: $HAuCl_4$, 1,4-NADH and NRase. The absorbance attributed to the NRase (strongest from 400-500 nm) becomes more pronounced as the NRase concentration is increased. **B)** Spectra of the same solutions recorded after 16 h (solutions in 1.5 mL plastic tubes at room temperature, no mixing).

S.2.4 Gold NP Size Calculation

The wavelength of the maxima of the surface plasmon resonance peak (λ_{max}) is often used as indication of the average NP size, with the broadness of the peak reflecting the NP uniformity (i.e. a broader peak has a large range in sizes of NPs). However, as the average NP size decreases, the environment around the NP becomes a more influential effect and thus using the λ_{max} alone would not give an accurate estimation of NP size. An improvement for estimating the NP size is described by W. Haiss et. al. and uses the absorbance at the surface plasmon resonance peak (A_{SPR}) and divides this by the absorbance at 450 nm (A_{450}).[2] The resulting ratio number is then used to estimate the average NP diameter using the equation:

$$d = exp\left(B_1 \frac{A_{SPR}}{A_{450}} - B_2\right)$$

where the experimentally determined fit parameters, B_1 = 3.00 and B_2 = 2.20 can be used. The authors determine there to be an approximately 11% standard deviation with this calculation method and do warn that it becomes less accurate for smaller NPs (< 10 nm). Thus, for these results, shown in **Table S 3**, where the NPs are calculated to be in the 2-5 nm range these should be considered an estimation which is more useful as a demonstration of the general trend of using higher NRase concentrations leading to smaller NPs than for giving highly accurate NP sizes. The TEM analysis is also used to confirm that this is a suitable estimate to make.

NPs below 2 nm become difficult to estimate their size since this is around the limit for NPs to give an absorbance in the UV-vis.[13] Thus, with the highest concentrations of NRase there may be very small Au NPs or nanoclusters (< 2 nm) which are not detected by UV-vis.

Table S 3. Analysis of the average NP size estimated from UV-vis spectra of reactions using different concentrations of NRase.

[NRase] (mg mL^{-1})	λ_{max} (nm)	A_{SPR} (a.u.)	A_{450} (a.u.)	A_{SPR} / A_{450}	d (nm)
0	-	-	-	-	-
0.4	530	0.61196	0.47158	1.29767	5.43578
0.8	524	0.87143	0.67868	1.28401	5.21750
0.8	523	0.88622	0.70076	1.26466	4.92337
1.5	516	0.70014	0.62897	1.11315	3.12507
1.9	513	0.55173	0.55247	0.99866	2.21664
1.9	514	0.54682	0.56275	0.97170	2.04438
1.9	513	0.55173	0.55432	0.99532	2.19454
2.9	-	-	-	-	-
3.6	-	-	-	-	-

S.2.5 Gold NP Size Distribution from TEM

After TEM images had been obtained, NP size analysis was carried out using ImageJ using the following protocol:

- Image threshold is adjusted to capture all NPs (**Figure S 4** and **Figure S 5**).
- NPs which can be seen in the image to be overlapping are removed so they do not skew the results, see **B**.
- NPs are analysed using ImageJ function.
- The size and circularity threshold are adjusted to only capture the NPs and not the background. For example, in the case of **Figure S 4**, size(nm^2): 1.70-Infinity and circularity: 0.10-1.00 were found to be good at capturing all the NPs while minimising the amount of background, see **C**.
- The area of each NP calculated by ImageJ is then used to calculate the diameter of each NP.
- The diameter data is then used in Origin to create statistical graphs (histogram or box plot) showing the range of NP diameters.

From these images, possible NPs of <1 nm were not included in the analysis since it was too difficult to distinguish between the background and these NPs. Thus, the first column in the size distribution histograms (**Figure S 6**) shows the number of particles between 1.0 to 1.5 nm size. The TEM analysis verifies the results acquired by UV-vis analysis – with the higher concentration of NRase leading to smaller sized NPs.

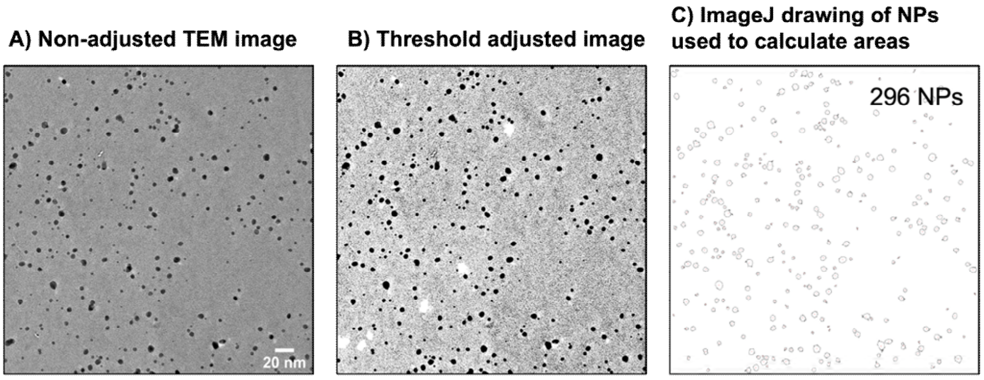

Figure S 4. TEM and NP size analysis for Au NPs made using 0.8 mg mL^{-1} NRase.

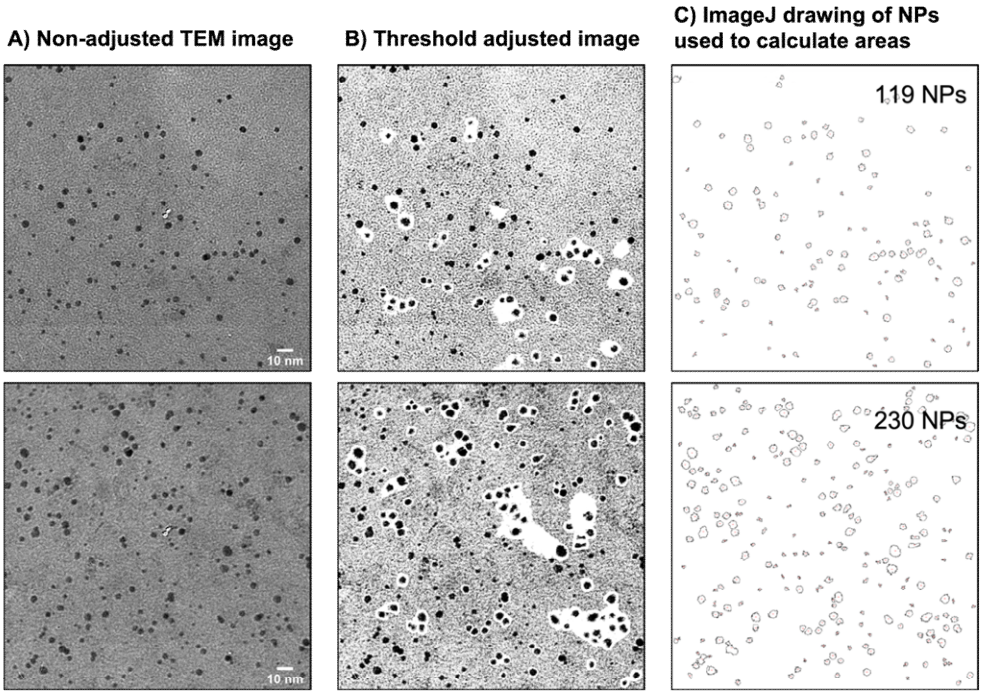

Figure S 5. TEM and NP size analysis for Au NPs made using 1.9 mg mL^{-1} NRase (the two images and subsequent analysis were combined to analyse a greater number of particles).

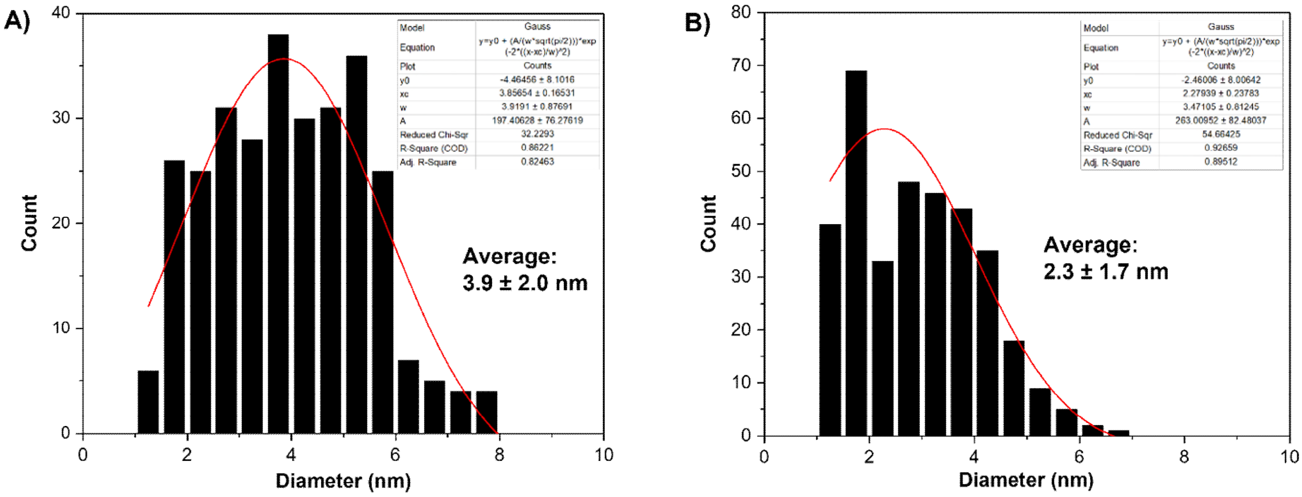

Figure S 6. Size distribution of Au NPs made using: **A)** 0.8 mg mL^{-1} and **B)** 1.9 mg mL^{-1} of NRase.

S.2.6 Gold NP Lattice Fringe Analysis

Good crystallinity of the Au NPs in the TEM images were observed. This allowed for lattice fringe analysis. **Figure S 7** shows the method used to estimate the d-spacing – in this example giving 0.283 nm as the measured lattice spacing which aligns with the interplanar distance expected for (111) plane of a *fcc* Au structure.[14] After Pt reduction has occurred the NPs are shown to retain their *fcc* crystallinity (**Figure S 8**).

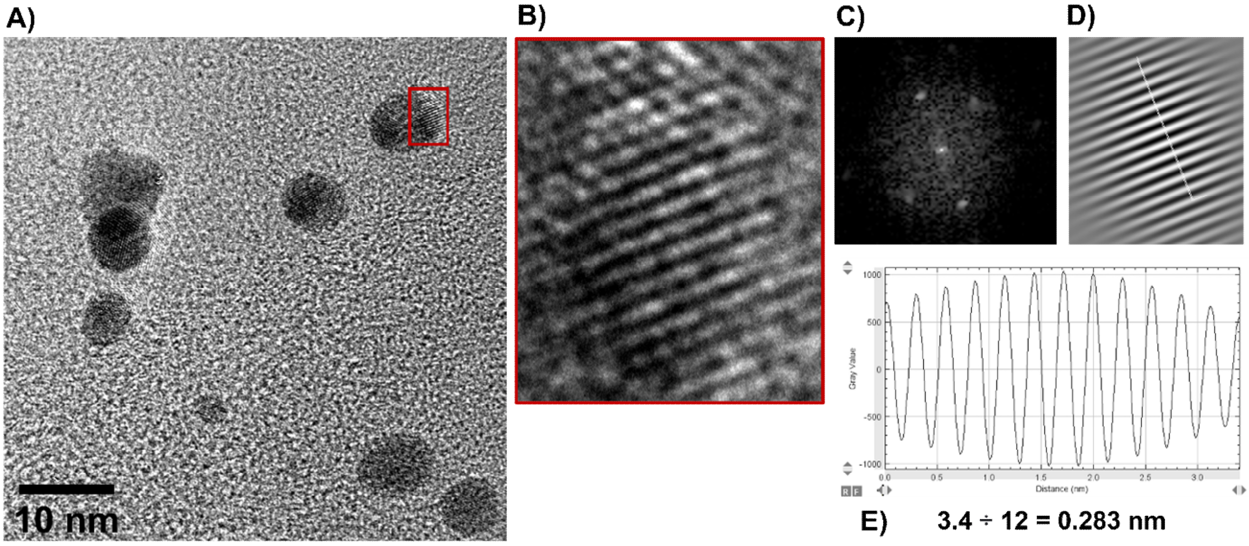

Figure S 7. Example of calculating the lattice spacing in Au NPs: these are *biohybrid NRase-Au NPs* (made using 0.8 mg mL^{-1} NRase). **A)** TEM image, red rectangle is area selected for the analysis. **B)** Zoomed-in section shown by the red rectangle in **A**. **C)** Fast Fourier transform (fft) of image **B**. **D)** Inverse fft with line drawn perpendicular to lattice fringes. **E)** Plot profile of line drawn in **D**, measuring the length of the line as 3.4 nm and there are 12 lattice fringes, therefore the average d-spacing across this line is 0.283 nm.

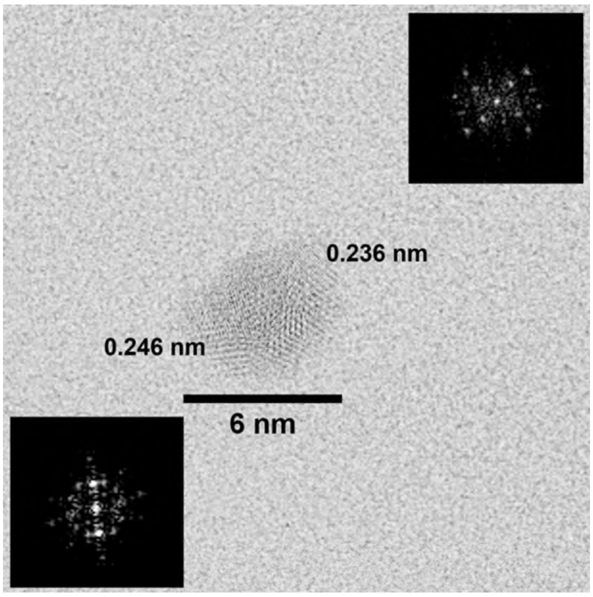

Figure S 8. HR-STEM BF image of *biohybrid NRase-Au@Pt NP* with insets of fast Fourier transform (fft) images showing lattice structure – spacing fitting with *fcc* lattice type. See **Figure S 16** for EDX analysis of the same NP.

S.2.7 Cofactor Studies

The native cofactor for the enzyme studied in this work, NRase, is 1,4-NADH. Another natural cofactor which is used by similar enzymes is 1,4-NADPH which only varies in the addition of a phosphate group (**Figure S 9** shows all the structures). Natural enzymes have evolved to be specific for either 1,4-NADH or 1,4-NADPH. There are a variety of non-natural cofactors, all containing the nicotinamide group, which can be made synthetically using a relatively simple and cheap method.[15-16] Two of these are used in this study – BNAH and AmNAH.

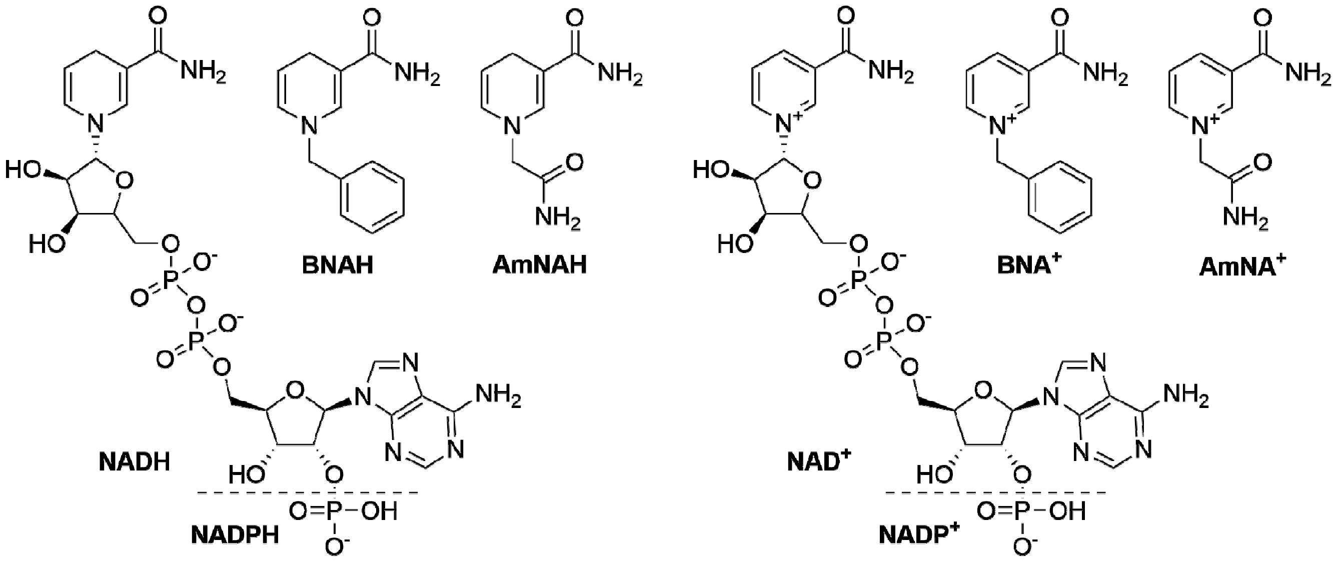

Figure S 9. Reduced and oxidised (left and right respectively) cofactor structures – the native cofactor for the NRase enzyme: NADH, the non-native NADPH and artificial cofactors: BNAH and AmNAH.

To confirm NRase causes the reduction of Au(III) and formation of Au NPs by oxidizing the supplied cofactor, 1,4-NADH, controls were carried out by using 1,4-NADPH instead of 1,4-NADH (**Figure S 10**). There is only a SPR peak for Au NPs in the UV-vis spectrum of the solution which contained $HAuCl_4$, NRase and 1,4-NADH. This indicates Au NP formation is catalysed by NRase by the hypothesised mechanism where NADH is oxidised at the buried active site (in which the specific nature of the configuration of amino acids surrounding the FMN does not allow for NADPH to undergo oxidation) and the resulting electrons can reduce the metal salt. The absorbance in the 400-600 nm region for the spectra of $HAuCl_4$, NRase and 1,4-NADPH is due to the absorbance from NRase.

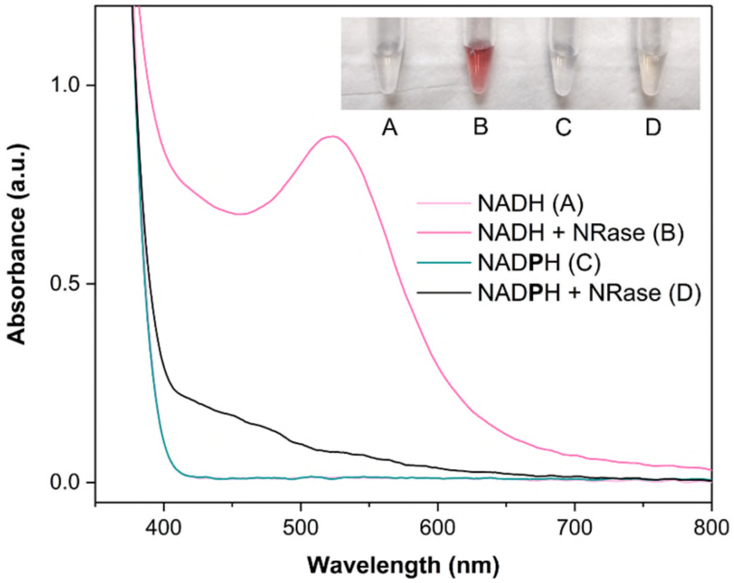

Figure S 10. UV-vis spectra of controls using the same conditions with either NADH or NADPH cofactor and in absence and presence of NRase. The inset shows a photograph of these solutions.

Next the artificial cofactors were tested and the results compared to NADH (**Figure S 11**). In the presence of the same concentration of NRase, the UV-vis spectra of the experiments using either NADH, BNAH or AmNAH, all have a similar λ_{max} (515 nm ± 1 nm) and a similar shape across 500-800 nm. Thus, indicating a similar size and shape distribution of Au NPs is made. This implies that the control of the NP morphology is directed by the NRase enzyme and not the cofactor. In the absence of NRase, while the UV-vis did not detect any Au NPs with NADH alone, there was significant absorbance across the 500-800 nm region when using BNAH or AmNAH cofactors. However the very broad nature of these absorbance peaks and the fact that precipitate was seen in these samples suggests these cofactors are able to reduce Au(III) but do not allow for size control or good stabilization of the resulting particles.

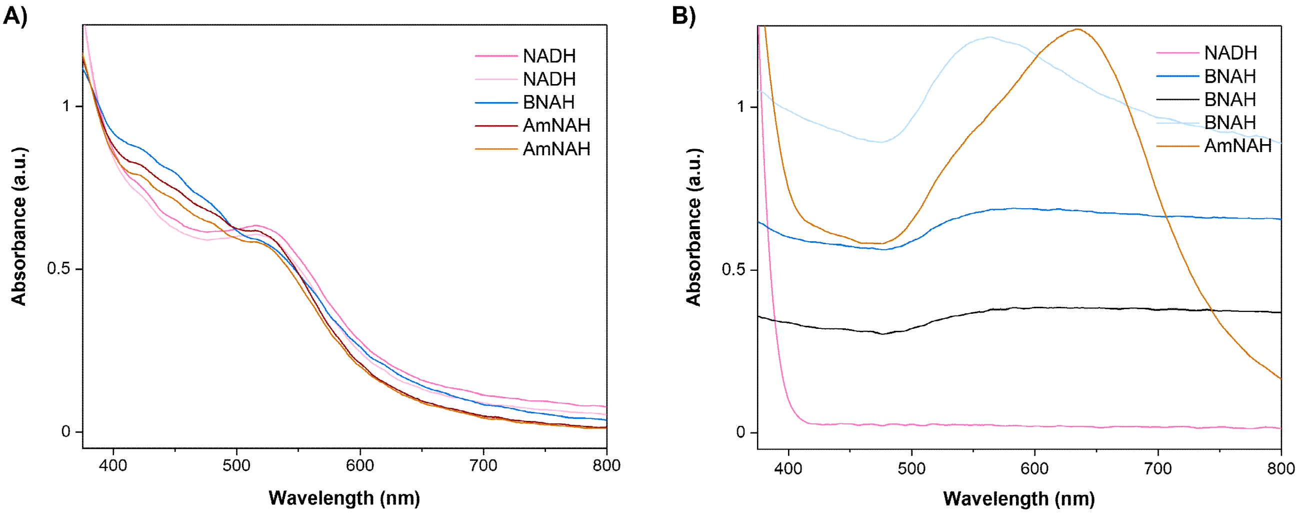

Figure S 11. UV-vis spectra of solutions containing a cofactor and $HAuCl_4$, after 16 h, **A)** in presence of NRase and **B)** in absence of NRase.

The TEM analysis confirmed the analysis made by UV-vis and showed the NPs made using NRase with NADH, BNAH or AmNAH were all spherical shaped NPs, under 10 nm in size (**Figure S 12**). The NPs prepared with BNAH without any NRase were seen to agglomerate and individual particles were seen to be irregular in shape and have a range of sizes.

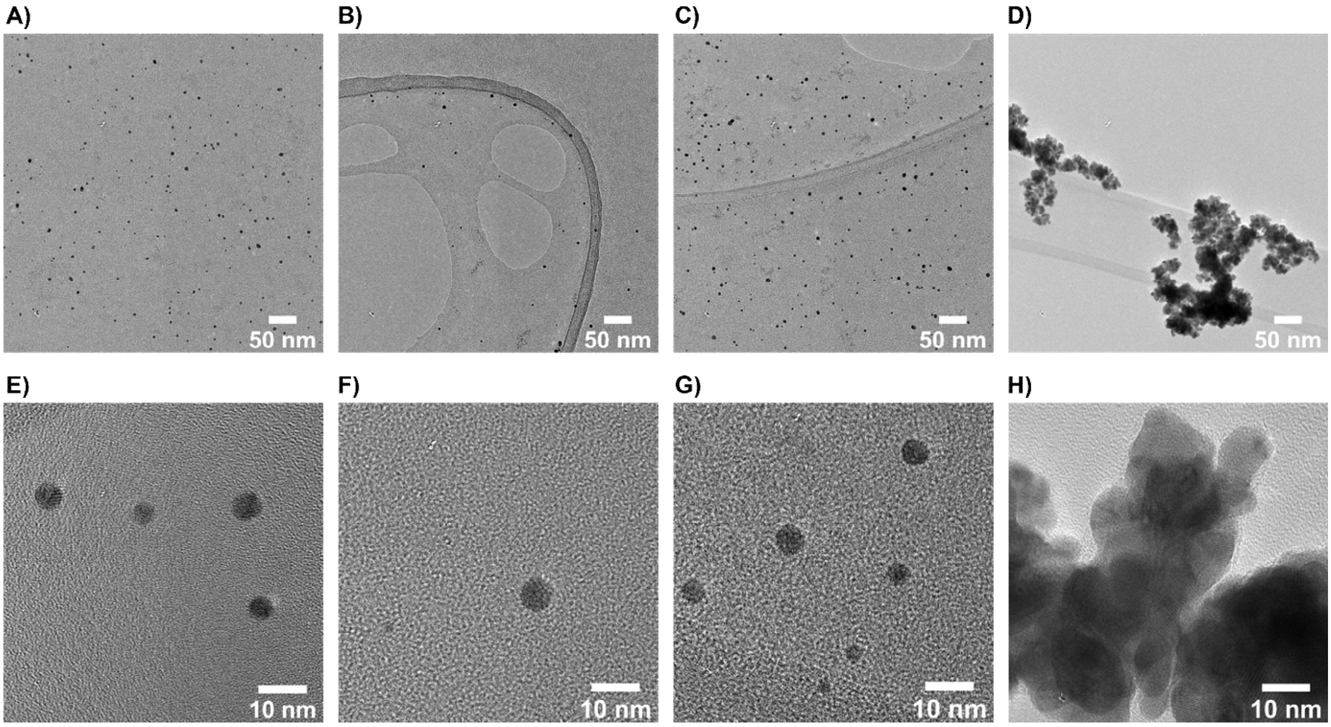

Figure S 12. TEM images from cofactor studies: **A)** & **E)** NADH with NRase, **B)** & **F)** BNAH with NRase, **C)** & **G)** AmNAH with NRase and **D)** & **H)** BNAH with no NRase. **A-D** lower magnification, **E-H** higher magnification.

The final experiment to verify that the whole NRase structure is required for achieving maximum NP size control and stability, involved testing FMN (the molecule where the hydride transfer occurs in the active site of NRase) and bovine serum albumin (BSA, a protein with no redox sites). The results using NADH as the cofactor (**Figure S 13**, **A**) showed only NRase could give good size control over the NPs, as indicated by the narrow SPR peak in the UV-vis spectrum. Whereas, replacing NRase for FMN (at approximately the same concentration) was found to, as expected, reduce Au(III) but with a very broad SPR peak indicating a large size/shape distribution of NPs. With FMN and BSA together there is an improvement in the shape of the absorbance spectrum compared to with FMN alone however much fewer NPs are formed thus BSA appears to limit the NP formation. BSA alone does not show any SPR absorbance confirming this is a suitable non-reactive protein to use as a control. The results using the artificial cofactor, BNAH, (**Figure S 13**, **B**) show a similar trend to the results with NADH. However, as it was shown earlier that BNAH alone can reduce Au(III) (**Figure S 11**, **B**), in the presence of BNAH and BSA Au NPs also formed. All the conditions tested do not give as good a size control as with NRase.

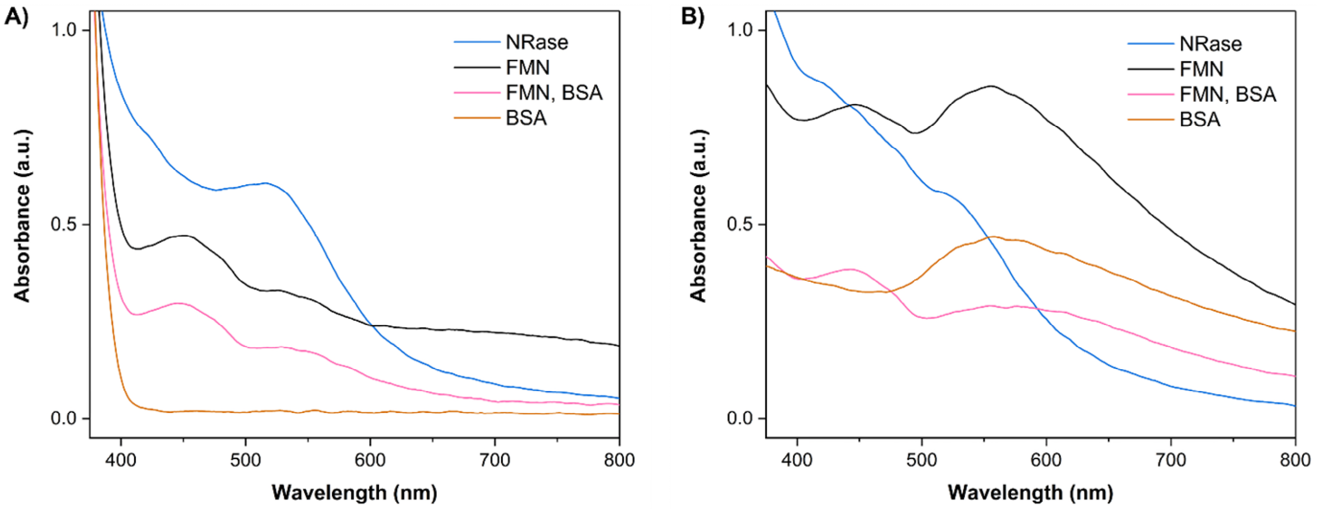

Figure S 13. UV-vis spectra of controls with FMN and/or BSA compared to NRase, after 16-17 h, **A)** with NADH cofactor and **B)** with BNAH cofactor.

S.2.8 Gold-Platinum NPs Size Distribution from TEM

The *biohybrid NRase-Au@Pt NPs* made by adding K_2PtCl_6 and NADH to a solution of already prepared Au NPs (made using NRase and NADH), were analysed by TEM. These were found to be quasi-spherical NPs of 6.4 ± 2.4 nm size (**Figure S 14**). The NPs prepared without NRase, when analysed by TEM, were found to contain a lot of large agglomerates and individual particles ranged in sizes – size distribution could not be accurately measured due to the overlapping nature of the NPs in the TEM images (**Figure S 15**).

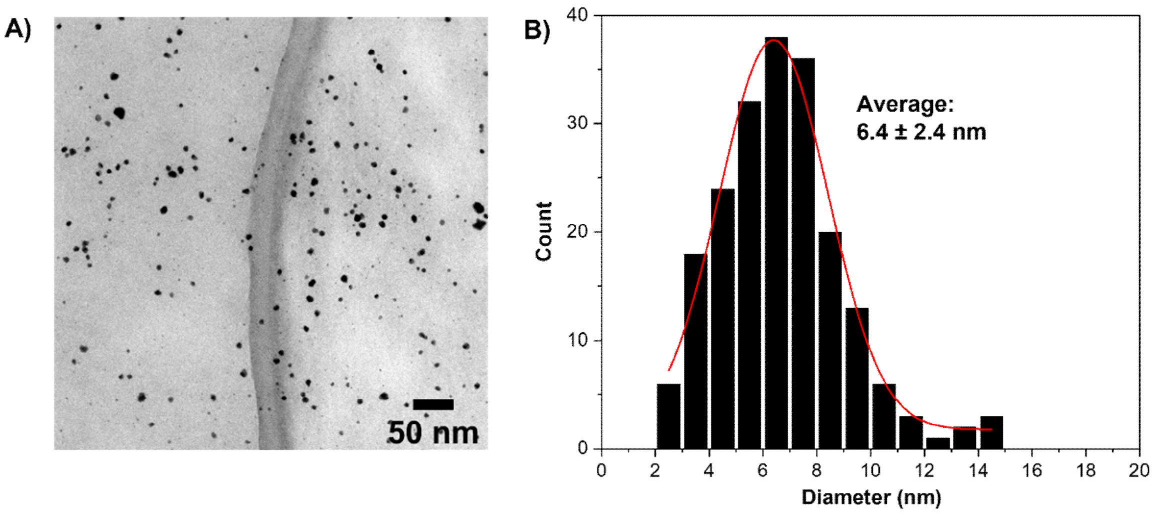

Figure S 14. Example of TEM analysis of *biohybrid NRase-Au@Pt NPs*, **A)** TEM image and **B)** NP size analysis (of 202 NPs, ignoring NPs smaller than 2 nm).

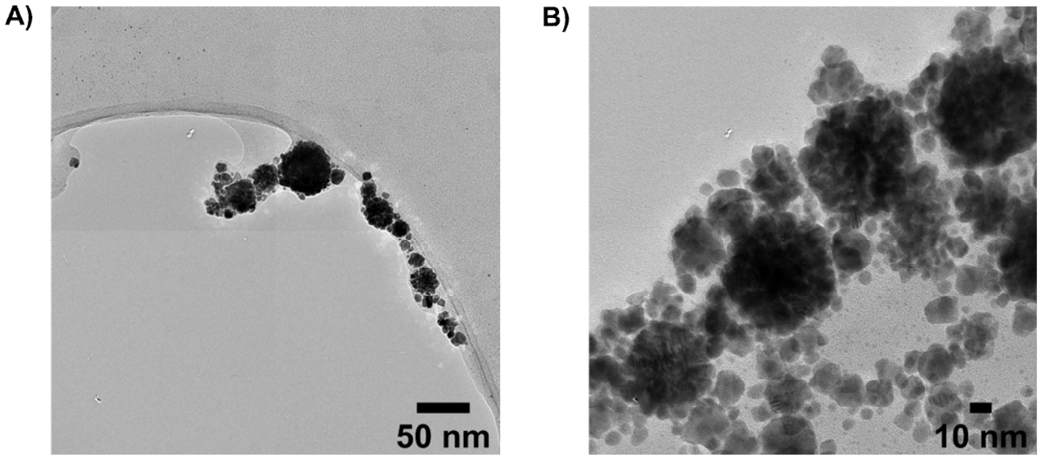

Figure S 15. TEM images of *non-bio-Au-Pt NPs* (Au-Pt NPs prepared without using NRase).

S.2.9 Gold-Platinum NPs HR-STEM-EDX Analysis

The following uncertainties should be taken into consideration when analysing these results: an EDX signal is always going to be somewhat delocalised due to the interaction volume of the electrons with the sample and the relatively large escape depth for x-rays and there may be some deformation of the nanoparticle under the beam. For the analysis the HR-STEM HAADF images of the corresponding NPs were used to measure the NP diameter and the position of the NP along the EDX line scan. The EDX line scan data was “smoothed” in Origin using a “Savitzky-Golay” method, with 20 pts of window and a polynomial order of 1. The shell thickness was estimated by using the distance from the edge of the NP (determined using the HAADF image, ± 0.1 nm) to the point at which the Au and Pt lines cross.

Thus, to calculate the core and shell thickness of the *biohybrid NRase-Au@Pt NPs* (made using NRase and the two-step procedure), the distances shown in **Figure S 16**, **A** were applied to the HR-STEM-EDX line scans shown in **Figure S 16**, **B** and **C** and therefore used to calculate the Au core diameter and Pt shell diameter. Using averages from the two line scans therefore calculated the Au core as 3.8 ± 0.1 nm and the Pt shell as 0.9 ± 0.4 nm.

The *non-bio-Au-Pt NPs* (made without NRase, with the same 2-step procedure), showed structures with more of an alloy composition or a Pt-core Au-layer and thin Pt-shell, as shown in **Figure S 17**, **A** and **B**. The *NRase-Au-Pt NPs,* prepared with NRase and with a 1-step procedure, also gave alloy structures and Pt-core Au-layer and thin Pt-shell structures such as those shown in **Figure S 17**, **C** and **D**.

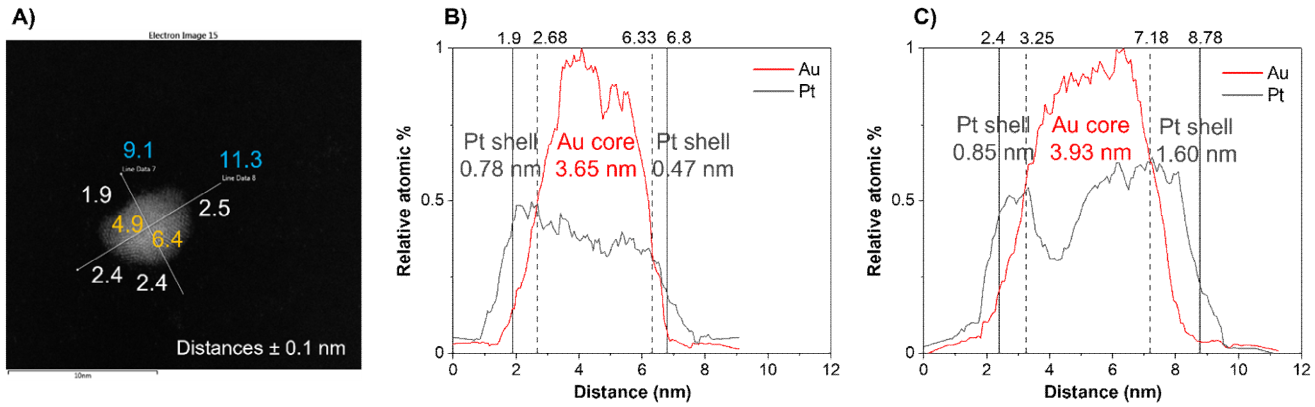

Figure S 16. EDX analysis of *biohybrid NRase-Au@Pt NP*, prepared using NRase and a two-step procedure: **A)** HR-STEM HAADF image of NP showing two line scans with distances measured using ImageJ (blue numbers are total length of line scan, yellow are diameter of NP across line scan and white are distance from start or end of line scan and edge of NP along line scan). **B)** HR-STEM-EDX line scan showing data converted to relative atomic % of detected Au and Pt along the line scan distance (of line data 7, 9.1 nm total, as labelled in **A**. **C)** HR-STEM-EDX line scan showing data converted to relative atomic % of detected Au and Pt along the line scan distance (of line data 8, 11.3 nm total, as labelled in **A**.

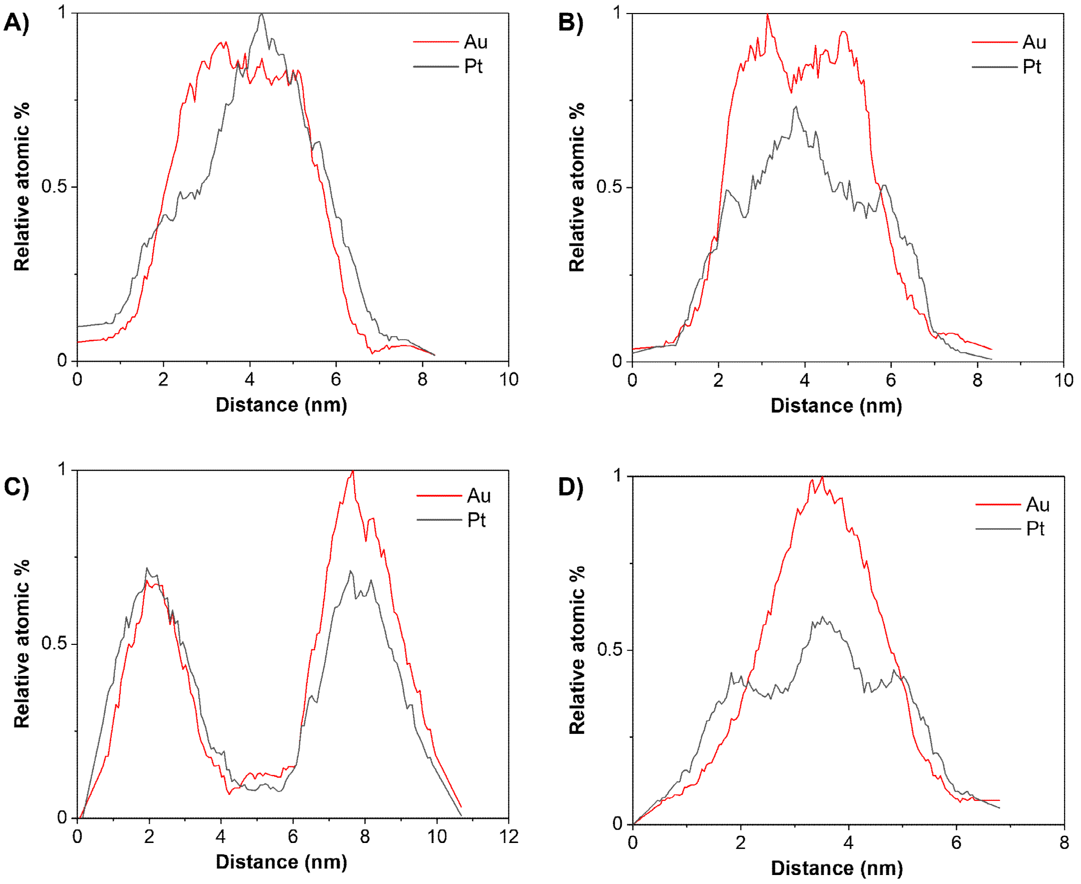

Figure S 17. EDX analysis of Au-Pt NPs: **A)** & **B)** examples of line scans from NPs prepared without NRase enzyme *(non-bio-Au-Pt NPs),* **C)** & **D)** examples of line scans from NPs prepared with NRase enzyme and with a 1-step procedure (*NRase-Au-Pt NPs*).

S.2.10 1-Step Versus 2-Steps for Gold-Platinum NP Formation

A comparison of using a 1-step procedure, where the Au(III) and Pt(IV) are added at the same time, versus the original 2-step procedure where Au(III) is added first and Pt(IV) is added after an initial 16 hours, was carried out. The resulting UV-vis spectra (**Figure S 18**), after both procedures have allowed the metals to be reduced for a total of 64 hours, appear to be very similar between the different methods used. However, from the HR-STEM-EDX data (see **S.2.9**) the structures do appear different with there being evidence for Au@Pt core-shell structures after the 2-step procedure whereas the 1-step procedure seems to give a mixture of Pt-core Au-layer and thin Pt-shell structures as well as alloy structures. Most interesting to note is that without NRase no NPs form after the initial 16 h, when Au(III) and Pt(IV) are added in either a 1-step or 2-step procedure (**B** and **D**) but after more time NPs do start to form as observed by the broad absorbance from 400-800 nm at 48 and 64 h time points.

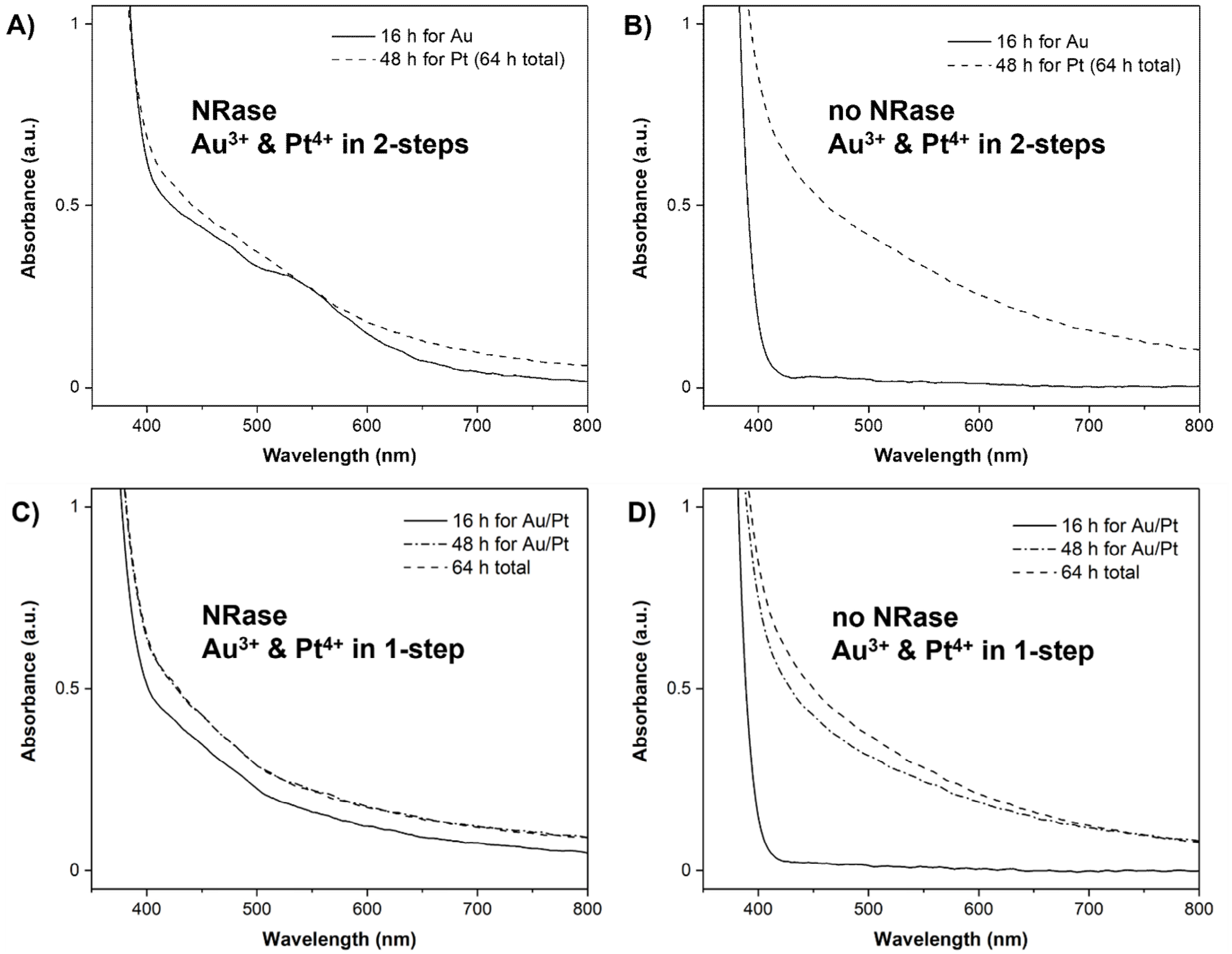

Figure S 18. UV-vis spectra recorded at different time points after $HAuCl_4$ and K_2PtCl_6 were either added in '2-steps' (with $HAuCl_4$ added first and K_2PtCl_6 after 16 h) or added at the same time, '1-step': **A)** 2-step addition with NRase present, **B)** 2-step addition with no NRase, **C)** 1-step addition with NRase present, **D)** 1-step addition with no NRase.

S.2.11 Control without Gold for Platinum NPs

A side-by-side experiment of four conditions in the absence and presence of gold and of NRase was carried out. The UV-vis spectra recorded at time points after K_2PtCl_6 and NADH were added are shown in **Figure S 19**. The very low absorbance seen in the spectra of the 'no Au' solutions (**C** and **D**) suggests only low concentration of Pt NPs were formed.

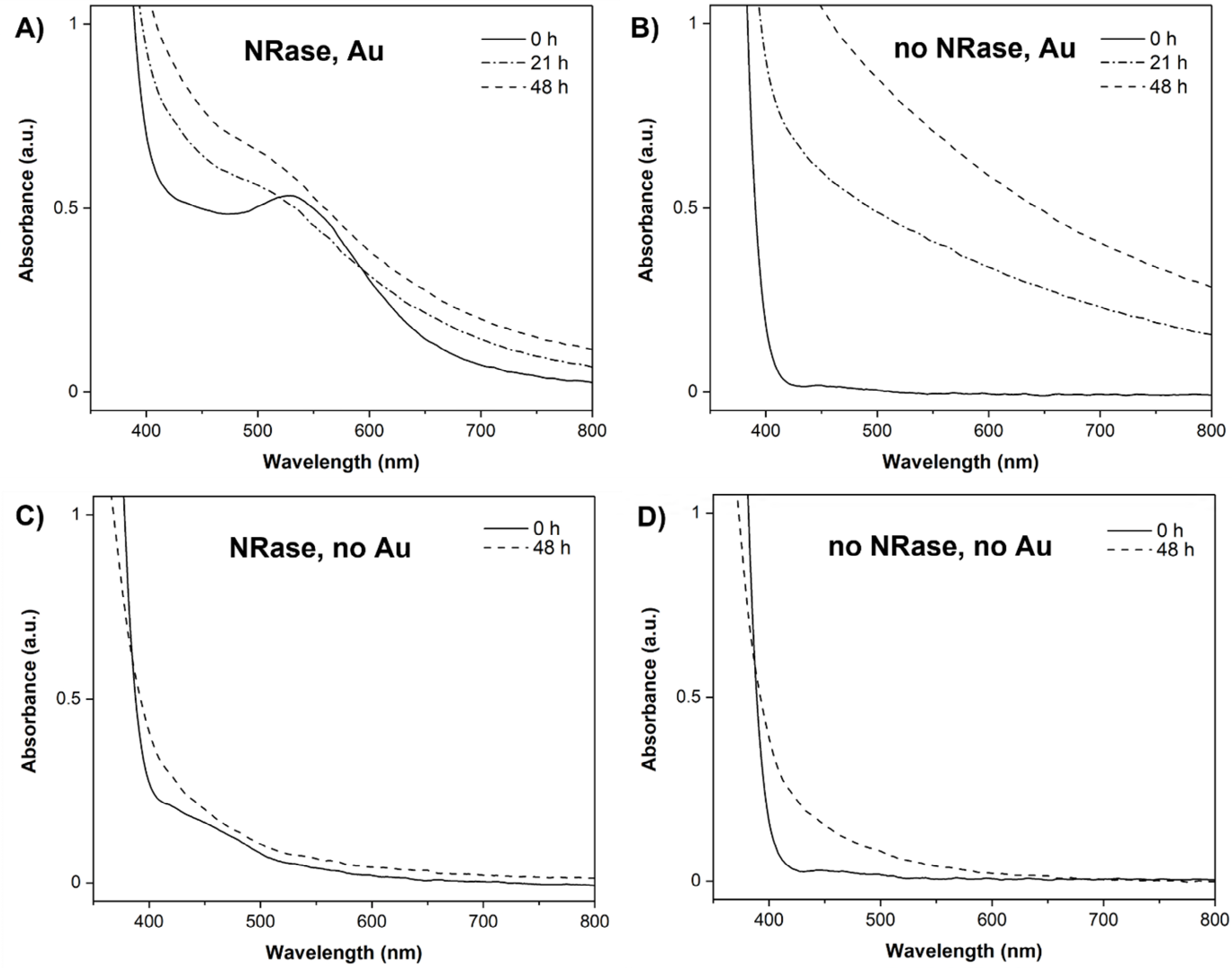

Figure S 19. UV-vis spectra recorded at different time points after K_2PtCl_6 and NADH were added to a solution which either contained: **A)** Au NPs made using NRase and NADH, **B)** no NRase but a solution of "aged Au salts and NADH", **C)** NRase but no Au, or **D)** no NRase and no Au. The "aged Au salts and NADH" solution means the same amount of $HAuCl_4$ and NADH as were used to make the Au NPs in **A** had been left under the same conditions (16 h, at room temperature).

S.2.12 Comparison of Using Different Ratios of Pt(IV) to Au(III)

The effect of varying the ratio of Pt(IV) to Au(III) on the synthesis of Au-Pt NPs was tested by either using a slight excess of Au or a slight excess of Pt (**Figure S 20**). These tests were carried out using the '2-step' procedure where Au(III) is added first and Pt(IV) is added after an initial 16 hours. The difference between the results is most apparent for the conditions with no NRase where the absorbance is much larger for the sample using an excess of Pt – see **B** compared to **D**. The difference is more difficult to detect by UV-vis in the case of the conditions where NRase was present – **A** and **C**.

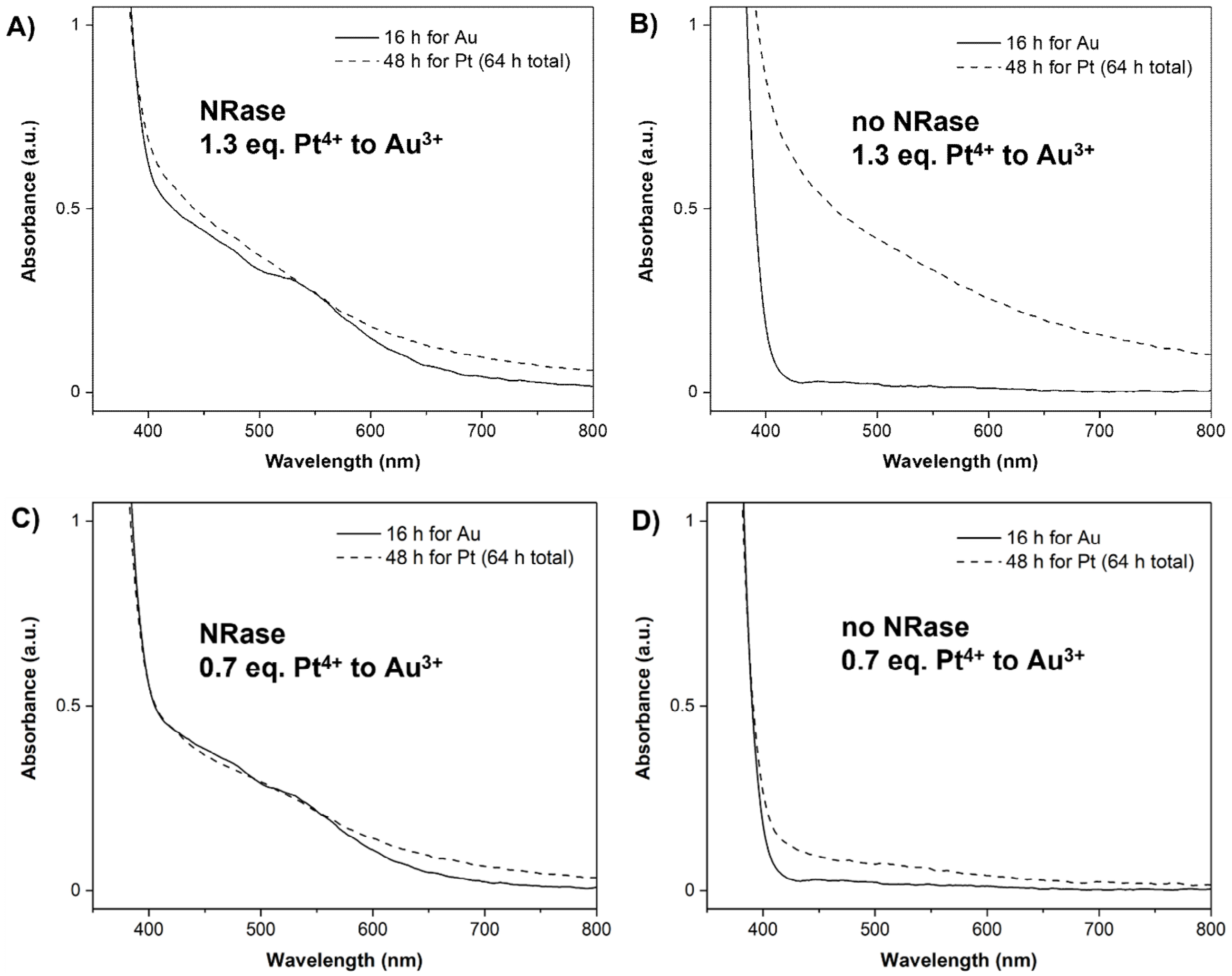

Figure S 20. UV-vis spectra recorded after 16 h from which $HAuCl_4$ and NADH were added together, followed by the addition of different amounts of K_2PtCl_6 and NADH, '16 h for Au', and then after leaving for a further 48 h, '48 h for Pt (64 h total)'. **A)** an excess, 1.3 molar equivalents, of Pt^{4+} to Au^{3+} was used, with NRase present, **B)** same conditions as **A** without NRase, **C)** 0.7 molar equivalents, of Pt^{4+} to Au^{3+} was used, with NRase present, **D)** same conditions as **C** without NRase.

S.2.13 Monitoring the Rate of Au NP Formation

The 16-hour reaction time used for testing different conditions in the synthesis of Au NPs was used to make sure each synthesis had reached completion. However as was seen by the colour change of the solutions, most were probably completed in a much shorter time. To investigate this, an experiment was performed by in situ monitoring of the reaction solution by UV-vis, recording spectra every 30 minutes (**Figure S 21**). From 0 to 1 hour, a fast initial rate of NP formation is observed by the increase at 509 nm (which was the final SPR λ_{max}) which is followed by a slower period where the absorbance at 509 nm decreases by 18%. This decrease in absorbance may be related to the FMN in the active site of NRase becoming reduced to $FMNH_2$ once there are no more metal salts for $FMNH_2$ to reduce by itself being oxidized. FMN has an absorbance peak with λ_{max} at 445 nm which decreases as is it reduced to $FMNH_2$.[17]

Another factor worth mentioning that has not been investigated thus far is the effect of stirring as all the NP synthesizes have been carried out without any stirring. Thus, the rate of NP formation is likely to be increased by introducing stirring.

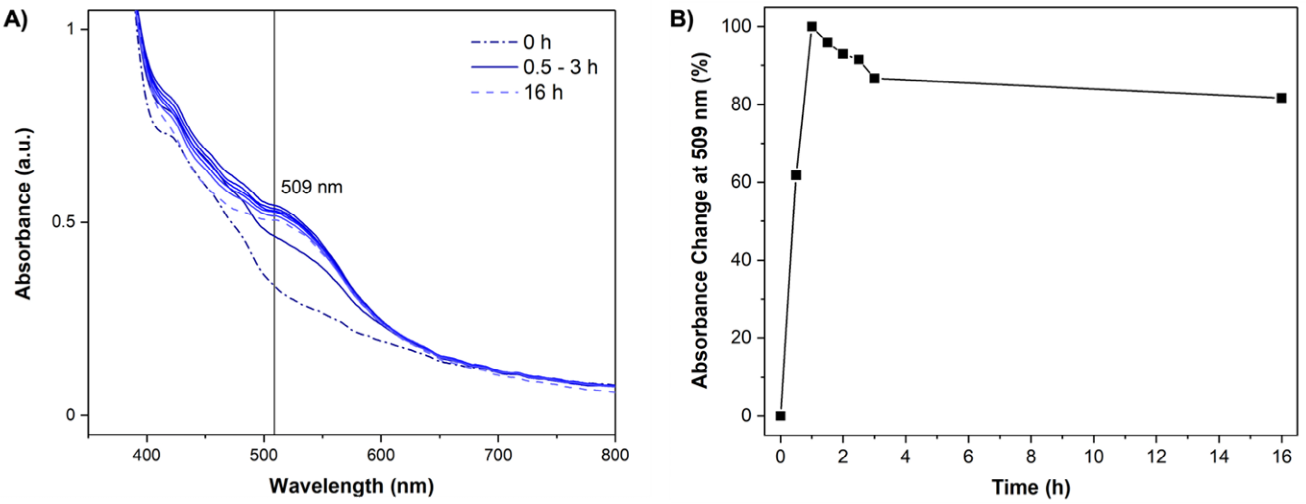

Figure S 21. Monitoring the synthesis of Au NPs using UV-vis spectrometry. **A)** UV-vis spectrum recorded immediately after addition of all reagents: $HAuCl_4$, 1,4-NADH and NRase (0 h) and then subsequent spectra recorded every 30 mins for 3 h, then a final spectrum recorded after 16 h. The formation of Au NPs is apparent within 30 minutes and at 1 h the SPR peak becomes more defined with the peak maximum at 509 nm. **B)** Plot of the absorbance change at the λ_{max} (509 nm) over time, with respect to the maximum absorbance at 509 nm which was recorded at 1 h.

S.2.14 NAD$^+$ Reduction Studies

The reduction of NAD$^+$ can lead to different products depending on the catalyst and conditions used. As described in the main text, 1,4-NADH is the only bioactive cofactor out of the possible products, shown in **Figure S 22**. Therefore, it is important to study the selectivity for 1,4-NADH.

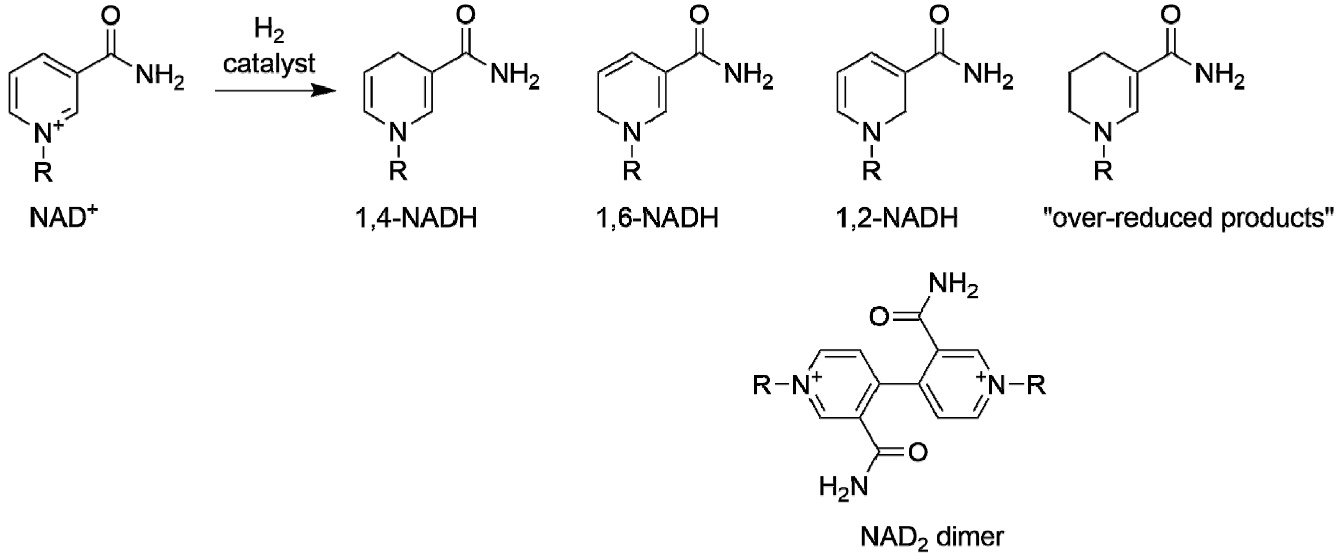

Figure S 22. NAD$^+$ reduction and potential products (R = adenosine diphosphoribose).

For NAD$^+$ reduction using H_2 and Au-Pt NPs, the following conditions were tested: the amount of NRase used in the NP synthesis, the ratio of Au^{3+} to Pt^{4+} used in the NP synthesis, whether the Au^{3+} and Pt^{4+} salts were added at the same time or not ('1-step' versus '2-step'), as well as adding more NRase after the NP synthesis for the NAD$^+$ reduction. A few recycling tests were also attempted. All results, with analysis after a reaction time of 21 h, are shown in **Table S4**.

The results from entries 1-7 and -10 show that as the amount of NRase in the NP synthesis is increased, there is an increase in selectivity for 1,4-NADH, while the conversion decreases. Entries 7 and 8 show that using a slight excess of Pt compared to Au is better for conversion of NAD$^+$ than an excess of Au to Pt. Entries 7 and 9 show that using the '1-step' procedure for making Au-Pt NPs may

be detrimental for achieving conversion of NAD^+. Adding more NRase after the NP synthesis gave improvement in results with all batches of NPs tested (comparing entry 1 with 11, entry 3 with 12 and entries 4-5 with 13). The amount of NRase added to these preformed NPs was less than used in the NP synthesis (e.g. 0.54 mg mL^{-1} NRase in NP syn. equates to 0.16 mg mL^{-1} once added to a solution of NAD^+, while the concentration of extra NRase added to this solution is 0.07 mg mL^{-1}). Thus, suggesting some of the NRase after the Au-Pt NP synthesis is no longer in an active form, perhaps due to being involved in the stabilisation of the NPs.

The preliminary studies of recycling the *biohybrid NRase-Au@Pt NPs* were carried out by filtering the organic reaction products through a size exclusion membrane (10 kDa, 1 nm pore size) and thus reusing the NPs and enzyme left behind in the filter for a second and subsequently a third reaction. The results (entries 12, 14 and 15) suggested the enzyme remained active, with no loss in selectivity across a total of 63 hours. There was however loss in total conversion – likely due to a non-optimized recycling procedure where some NPs and enzyme may be lost between reactions, on the membrane filter. Further studies are needed to assess the recyclability of these NPs for repeated batch or flow systems.

Table S 4. Results from H_2-driven, NAD^+ reduction studies using *non-bio-Au-Pt NPs, NRase-Au-Pt NPs* and *biohybrid NRase-Au@Pt NPs*, prepared with different NRase concentrations.[a]

Entry	NP synthesis conditions:			[NRase] in NAD^+ reaction (mg mL^{-1}):		Selectivity for 1,4-NADH (%)[c]	Conversion of NAD^+ (%)[d]
	NP batch[b]	[NRase] in total NP syn. (mg mL^{-1})	Au^{3+}: Pt^{4+} molar ratio	Introduced during NP syn.	Added after NP syn.		
1	1a	0	1: 1.1	0	0	0	>99
2	1b	0.20	1: 1.1	0.06	0	17	>99
3	2b	0.54	1: 1.1	0.16	0	64	88
4	2c	0.81	1: 1.1	0.24	0	74	33
5	2c	0.81	1: 1.1	0.24	0	73	37
6	3b	1.88	1: 1.1	0.55	0	>99	20
7	4b	1.60	1: 1.3	0.47	0	>99	41
8	4c	1.60	1: 0.7	0.47	0	N/A	0
9	4d	1.60	1: 1.3[e]	0.47	0	N/A	0
10	4e	3.19	1: 1.3	0.93	0	N/A	0
11	1a	0	1: 1.1	0	0.07	96	>99
12	2b	0.54	1: 1.1	0.16	0.07	>99	>99
13	2c	0.81	1: 1.1	0.24	0.07	>99	>99
14[f]	2b	0.54	1: 1.1	0.16	0.07	>99	68
15[g]	2b	0.54	1: 1.1	0.16	0.07	>99	40

[a] Reaction conditions follow **S.1.7.1**. Analysis after reaction time of 21 h. [b] Different batches of NPs (number and letter referring to experiment no. and sample no. respectively: “a” indicates no NRase used). [c] Selectivity is measured by the content of 1,4-NADH as a percentage out of the total reaction products, as analysed by ^1H NMR spectroscopy. [d] Conversion measures the consumption of NAD^+ starting material, as analysed by ^1H NMR spectroscopy. [e] Au^{3+} and Pt^{4+} salts added together at same time, using the ‘1-step’ procedure. [f] Recycled NPs/enzyme study: after first reaction (entry 12), reaction solution filtered with 10 kDa size exclusion filter and new NAD^+ solution added. [g] Recycled NPs/enzyme study: after second reaction (entry 14), reaction solution filtered with 10 kDa size exclusion filter and new NAD^+ solution added.

In analysis by ^1H NMR, the 5.0 to 10.0 ppm spectral region is useful for quantifying reaction products (**Figure S 23**). As NAD^+ is reduced, the nicotinamide ring loses its aromaticity thus there is the disappearance of the peaks at high ppm (8.2, 8.8, 9.1 and 9.3 ppm) and the appearance of a single new peak in this region, at 6.9 ppm, indicates the 1,4-NADH isomer has been made. If there are other peaks in the 6.8 to 7.4 ppm region this indicates other NADH isomers, i.e. 1,6-NADH and 1,2-NADH, are present.

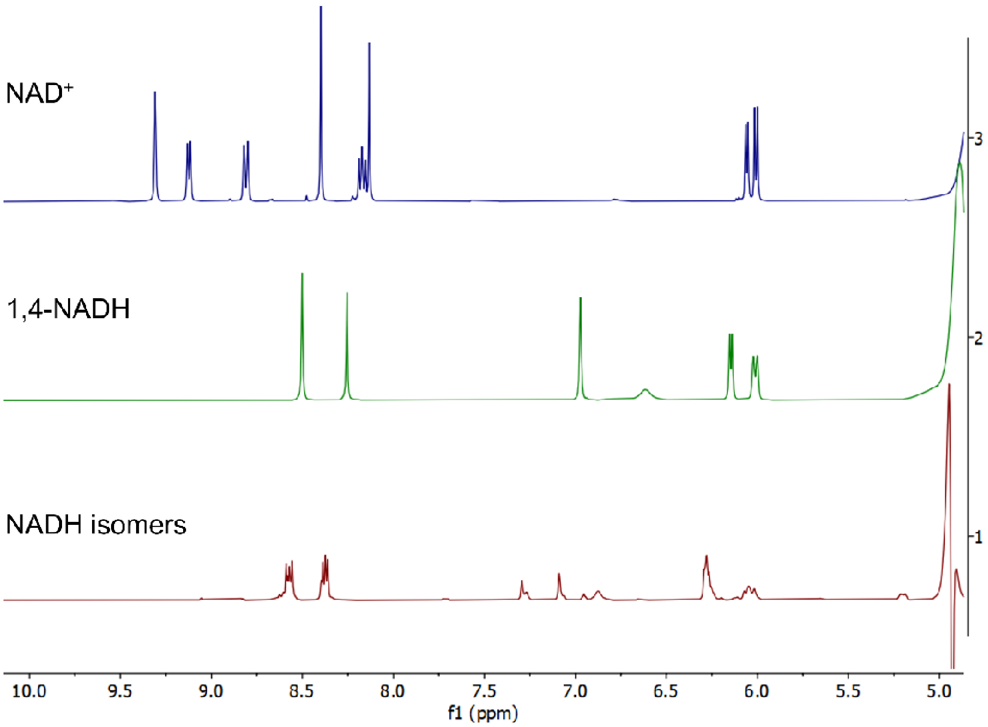

Figure S 23. ^{1}H NMR spectra (20% D_2O in aqueous 5 mM potassium phosphate pH 8) of standards of NAD^+ and 1,4-NADH and a mixture of other NADH isomers made by reacting NAD^+ with $NaBH_4$.

As well as other NADH isomers, in the reactions with the *non-bio-Au-Pt NPs* there is evidence for over-reduced products (i.e. further reduction of the nicotinamide ring). This can be seen by the peaks at 2.8 to 3.4 ppm indicating saturated bonds (**Figure S 24**).

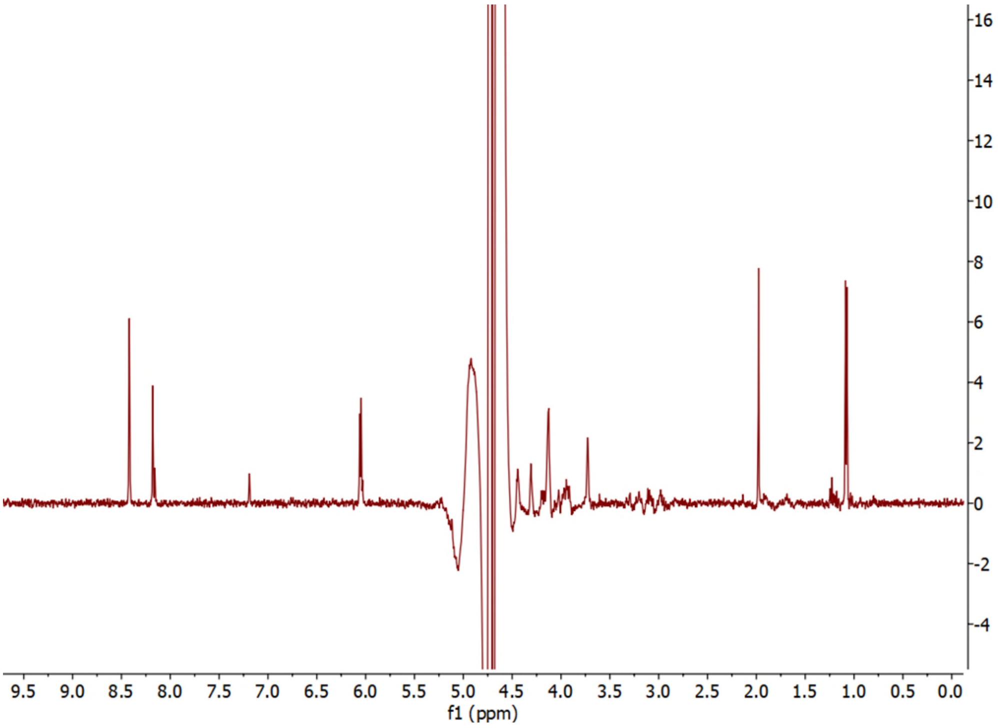

Figure S 24. ^{1}H NMR spectra (20% D_2O in aqueous 5 mM potassium phosphate pH 8) of example from NAD^+ reduction reaction using *non-bio-Au-Pt NPs* without any NRase (**Table S 4**, entry 1) giving no selectivity for 1,4-NADH and instead giving over-reduced products (as evidenced by the peaks at 2.8-3.4 ppm).

When NRase (in high enough concentration) is included in the NAD^+ reduction reactions, the ^1H NMR spectra show complete selectivity for 1,4-NADH isomer such as in the example shown in **Figure S 25**.

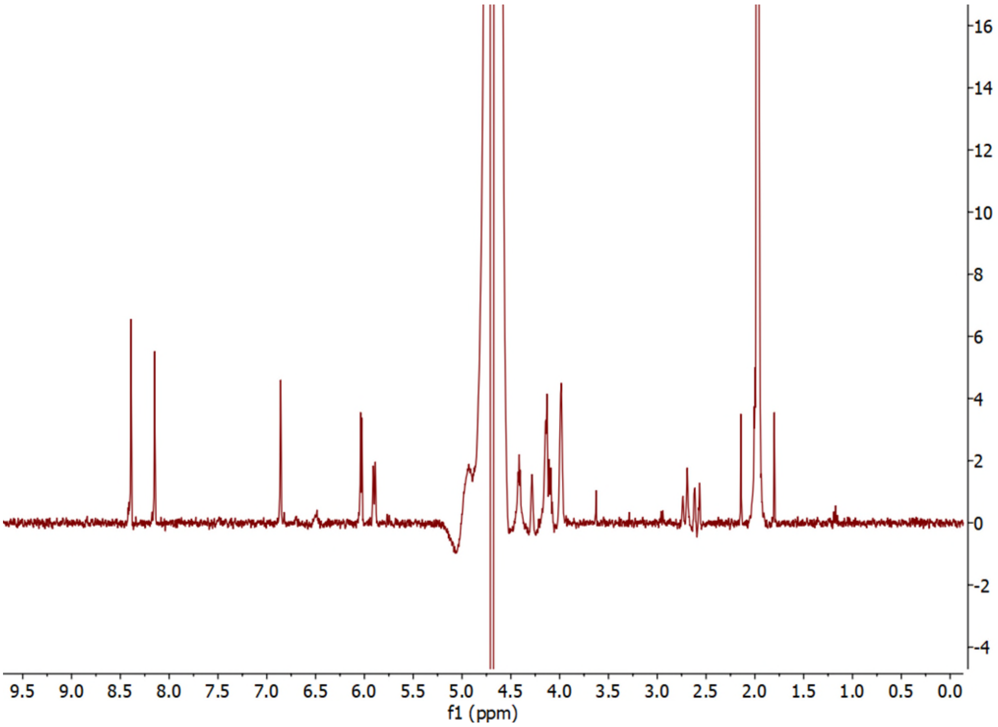

Figure S 25. ^1H NMR spectra (20% D_2O in aqueous 5 mM potassium phosphate pH 8) of example from NAD^+ reduction reaction using *biohybrid NRase-Au@Pt NPs* (**Table S 4**, entry 13) where conditions were selective for 1,4-NADH (with complete conversion of NAD^+).

S.2.15 4'-Chloroacetophenone Reduction

The results displayed in **Table S5** are from reactions comparing reaction conditions and the type of NPs that were used. The initial conditions tested were based on those from previous work in the group where a NADH dehydrogenase was coupled with a Pd/C catalyst.[18] After long reaction times, the conversion was moderate but not high (entries 1-2), thus since sulfur containing molecules are known to often coordinate strongly to metal NPs, the tris buffer and DMSO co-solvent was exchanged for no buffer (unbuffered water instead) and acetonitrile as the co-solvent to solubilize 4'-chloroacetophenone in solution. This change in conditions allowed for 91-98% conversion of product (entries 3-4). Due to the acidic nature of NAD^+ these reaction solutions were around pH 6 which may not be optimal for recycling the catalyst as pH 6 is also around the electrostatic potential (pI) of NRase meaning NRase can cause aggregation of the NPs by weakening stabilization forces. Indeed, after attempting to recycle the catalyst from these reactions, only very low conversion (<10%) was obtained. Therefore, a low concentration of a non-coordinating buffer was introduced, 3.5 mM of pH 8 potassium phosphate, which was enough to keep the pH at pH 8 (with 1 mM NAD^+ concentration). Shorter reaction times were also focused on.

As expected from the NAD^+ reduction experiments, without adding extra NRase, the conversions were significantly lower (entries 5-6). Increasing the amount of NRase added did not significantly increase the conversion (entries 8-9). However, it was later found that increasing the amount of metal NPs present did significantly increase the conversion such that high conversion (entries 11-13) could be obtained within the 21-hour reaction length.

Reactions were also tested with ethanol instead of acetonitrile as the co-solvent. However as can be observed by the reaction without any NPs or NRase (but still with ADH), 1 vol% ethanol gave 22% product (entry 16). In this case ADH is oxidising ethanol while reducing NAD^+ and thus subsequently allowing it to reduce 4'-chloroacetophenone using the generated 1,4-NADH. There is therefore a build-up of ethanal which is not recycled. It is a useful comparison to the results with NPs and NRase which therefore shows that the biohybrid NRase-NP system can provide faster reaction rates than the ethanol system.

Pt NPs prepared without Au (which only formed in low concentrations under the synthesis conditions) were also tested by adding much higher volumes of the synthesis solution – to try to reach a similar concentration as the Au-Pt NPs prepared under the same conditions. The Pt NPs prepared without NRase gave only a slightly higher conversion than the control without NPs (entries 15-16). The Pt NPs prepared in the presence of NRase gave 64% conversion (entry 14) however the *biohybrid NRase-Au@Pt NPs* gave 95% (entry 13)

under the same conditions and with using less of the synthesis solution. Therefore these experiments confirm that using Au to catalyse Pt(IV) reduction is beneficial for the application of the resulting NPs.

Reaction optimisation has not been carried out thus far, therefore the catalytic activity values (TTN, TOF and TN) displayed in the table should only be considered as an initial starting point and not as fully optimised rates for this reaction and catalyst system which is beyond the scope of this communication.

Table S 5. Results from 4'-chloroacetophenone reduction studies using Au-Pt NPs and NRase to recycle 1,4-NADH which is required by ADH to carry out the enantioselective reduction of the ketone to (*S*)-4'-chloro-1-phenylethanol.[a]

Entry	NP synthesis conditions:			Reduction reaction conditions:				Time (h)	Product (%)[e]	NRase TTN[f]	NRase TOF (h^{-1})[f]	NAD^+ TN[g]
	NP batch[b]	[NRase] in NP syn. (mg mL^{-1})	Au^{3+}: Pt^{4+} molar ratio	[NRase] introduced during NP syn. (mg mL^{-1})	[NRase] added after NP syn. (mg mL^{-1})	Au, Pt mol%[c]	Buffer and cosolvent conditions[d]					
1[h]	5b	0.85	1: 1.3	0.25	0.36	2.0, 2.6	A	64	41	309	5	2.1
2[h]	5a	0	1: 1.3	0	0.36	2.0, 2.6	A	64	20	256	4	1.0
3	5b	0.85	1: 1.3	0.25	0.36	2.0, 2.6	B[i]	64	>99	747	12	5.0
4	5a	0	1: 1.3	0	0.36	2.0, 2.6	B[i]	64	88	1124	18	4.4
5	2b	0.54	1: 1.1	0.16	0	2.0, 2.2	C	21	4	115	5	0.2
6	5a	0	1: 1.3	0	0	2.0, 2.6	C	21	2	N/A	N/A	0.1
7	none	N/A	N/A	N/A	N/A	N/A	C	21	0	N/A	N/A	0.0
8	2b	0.54	1: 1.1	0.16	0.36	2.0, 2.2	C	21	18	159	8	0.9
9	2b	0.54	1: 1.1	0.16	0.51	2.0, 2.2	C	21	22	151	7	1.1
10	5a	0	1: 1.3	0	0.36	2.0, 2.6	C[j][k]	21	25	319	15	1.3
11	5a	0	1: 1.3	0	0.36	4.0, 5.2	C[j]	21	84	1073	51	4.2
12	5a	0	1: 1.3	0	0.36	2.0, 2.6	C[j]	21	33	422	20	1.7
13	2b	0.54	1: 1.1	0.32	0.36	4.0, 4.4	D	21	95	643	31	4.8
14	5b*	0.85	0: 1	0.48	0.36	0, 7.8	D	21	64	350	17	3.2
15	5a*	0	0: 1	0	0.36	0, 7.8	D	21	29	371	18	1.5
16	none	N/A	N/A	N/A	N/A	N/A	D	21	22[l]	N/A	N/A	1.1

[a] Reaction procedure as described in **S.1.7.2**. Conditions, unless otherwise stated: NAD^+ (1 mM), 4'-chloroacetophenone (5 mM), ADH-105 (0.44 mg mL^{-1}), H_2 (1 bar), room temperature (20-25 °C). [b] Different batches of NPs (number and letter referring to experiment no. and sample no. respectively: "a" indicates no NRase used and * has no Au). [c] Mol% calculated from concentration of Au^{3+} and Pt^{4+} used in the NP synthesis and adjusted to reflect concentration in the reaction solution relative to 5 mM concentration of 4'-chloroacetophenone. [d] A: tris-HCl (50 mM, pH 8), DMSO (1 vol%), B: no buffer (pH 6), acetonitrile (1 vol%), C: potassium phosphate (3.5 mM, pH 8), acetonitrile (1 vol%) and D: potassium phosphate (3.5 mM, pH 8), ethanol (1 vol%). All other conditions are kept the same, unless otherwise stated, as follows: NAD^+ (1 mM), 4'-chloroacetophenone (5 mM), ADH-105 (0.44 mg mL^{-1}), H_2 (1 bar), room temperature (20-25 °C). [e] Conversion was calculated using ^1H NMR spectroscopy by using a calibration curve of known concentrations of mixed 4'-chloroacetophenone and (*S*)-4'-chloro-1-phenylethanol and using the ratio between their absolute integrations at 7.90-8.05 ppm and 7.30-7.50 ppm respectively (see **Figure S 28**). Chiral-GC was also used to confirm some of the conversions and to determine the enantioselectivity of the product (see **Figure S 30**). [f] NRase total turnover number (mol of 4'-chloro-1-phenylethanol per mol of NRase) and NRase turnover frequency (mol of 4'-chloro-1-phenylethanol per mol of NRase per hour) using total mol of NRase in reaction (i.e. NRase introduced during NP synthesis + NRase added after NP synthesis) and mol of product determined from analysis at end of reaction therefore TOF only an approximation. [g] NAD^+ turnover number (mol of 4'-chloro-1-phenylethanol per mol of NAD^+). [h] A higher amount of ADH-105 was used (0.87 mg mL^{-1}). However, under these same conditions, using NP batch 5b, with 0.44 mg mL^{-1} ADH-105 the conversion was similar (44%). [i] These conditions led to low conversions (<10%) when recycling the catalyst. [j] These were carried out using 2.5 bar H_2. [k] The same conditions at 1 bar H_2 gave a similar conversion (20%). [l] This product is formed since ADH has oxidised the ethanol (present as a cosolvent) while reducing NAD^+ to NADH which it can then re-oxidise while reducing 4'-chloroacetophenone.

S.2.16 4'-Chloroacetophenone Reduction Analysis

^{1}H NMR spectroscopy was used as the main method for analysis of the 4'-chloroacetophenone reduction reactions, with the peaks in the 7.3 to 8.1 ppm region used to calculate the conversion (**Figure S 26**, **Figure S 27**, **Figure S 28**).

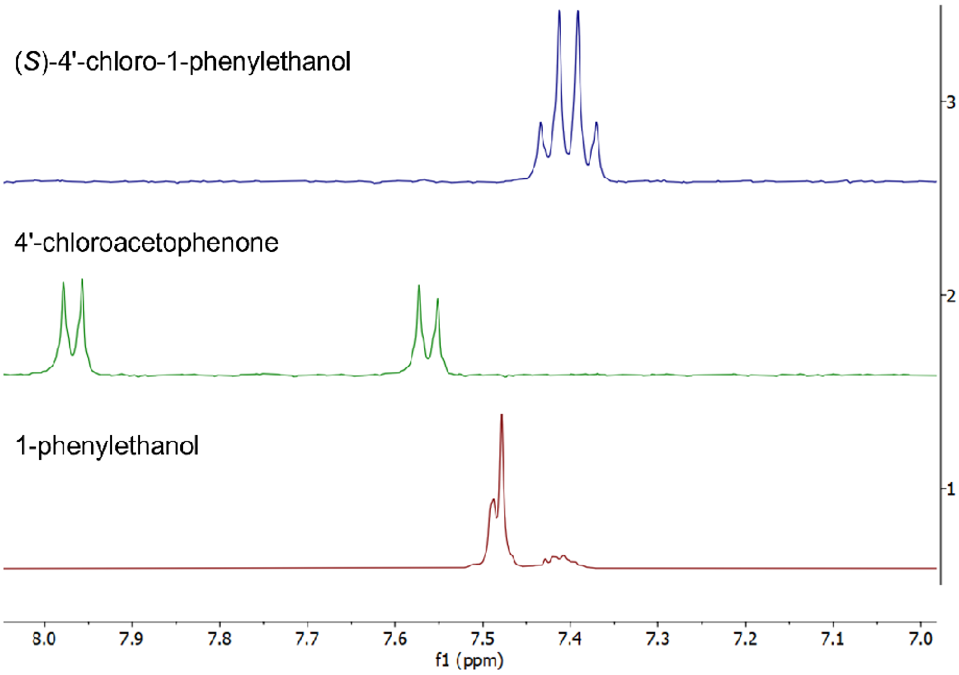

Figure S 26. ^{1}H NMR spectra (20% D_2O in aqueous 5 mM potassium phosphate pH 8) of standards of product, starting material and dechlorinated product (top to bottom spectra respectively).

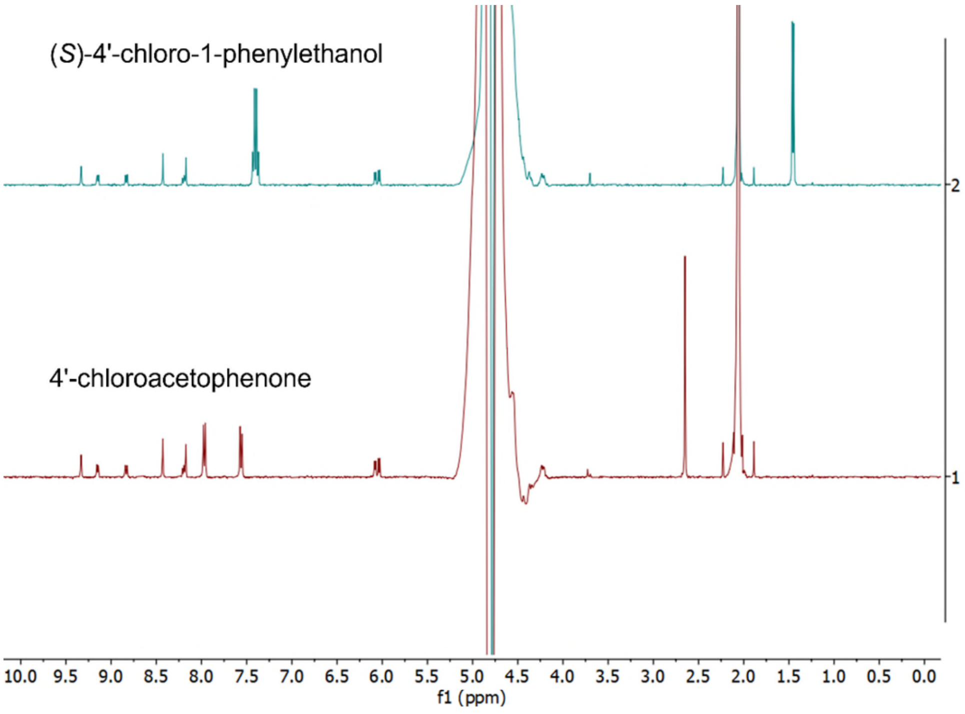

Figure S 27. ^{1}H NMR spectra (20% D_2O in aqueous 5 mM potassium phosphate pH 8) of standards of product and starting material in aqueous solutions (with 1 vol% acetonitrile) also containing 1 mM NAD^{+}.

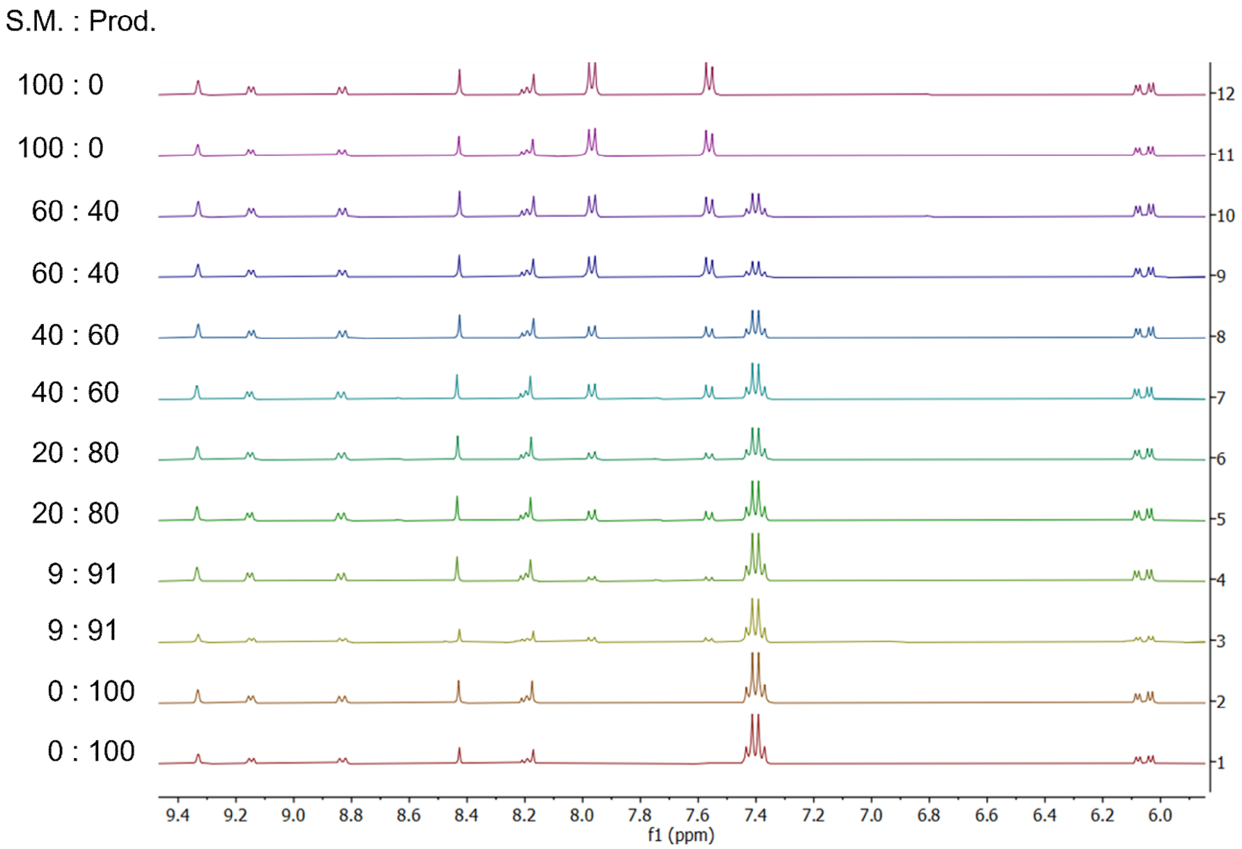

Figure S 28. ^{1}H NMR spectra (for calibration curve, showing 6.0 to 9.4 ppm region) of different ratios of starting material and product in aqueous solutions (with 1 vol% acetonitrile) also containing 1 mM NAD^{+}.

No dechlorinated products were observed in the ^{1}H NMR spectra of reactions. **Figure S 29** shows an example of a reaction result which had >99% product and <1% starting material remaining and no sign of a peak around 7.50 ppm which would indicate the presence of 1-phenylethanol.

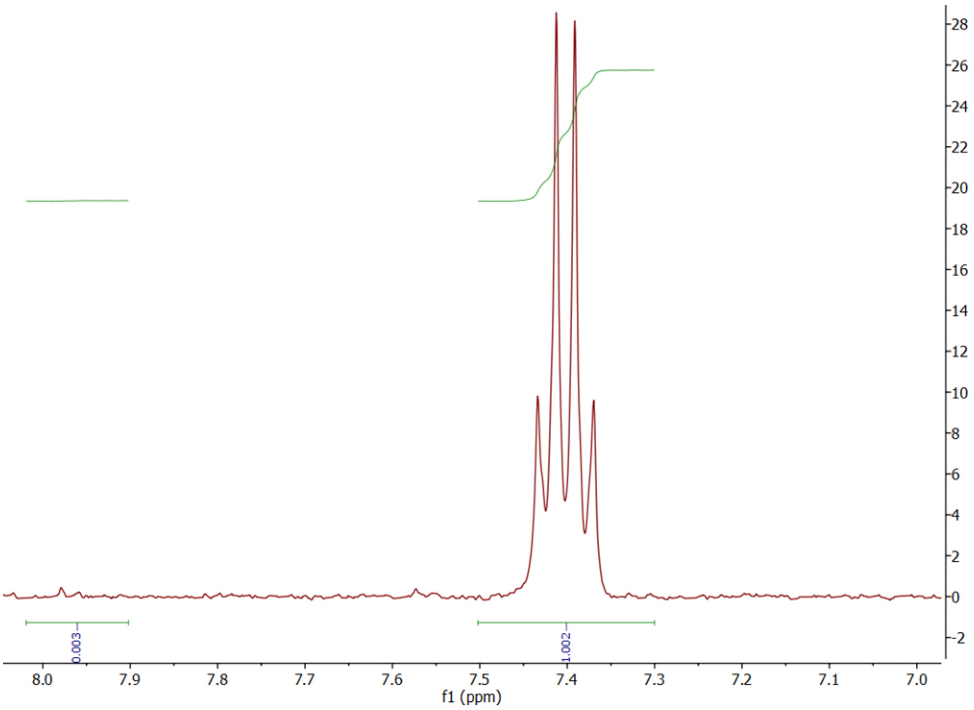

Figure S 29. ^{1}H NMR spectra (20% D_2O in aqueous 5 mM potassium phosphate pH 8), from 7.0 to 8.0 ppm, of example of reaction results (entry 3, >99% product). No dechlorinated product (1-phenylethanol) observed.

Chiral-GC analysis was also performed for selected samples to determine the enantioselectivity of the reaction (**Figure S 30**). The reaction conversion from the GC analysis correlated well with the NMR conversion results (up to 2% difference between the results).

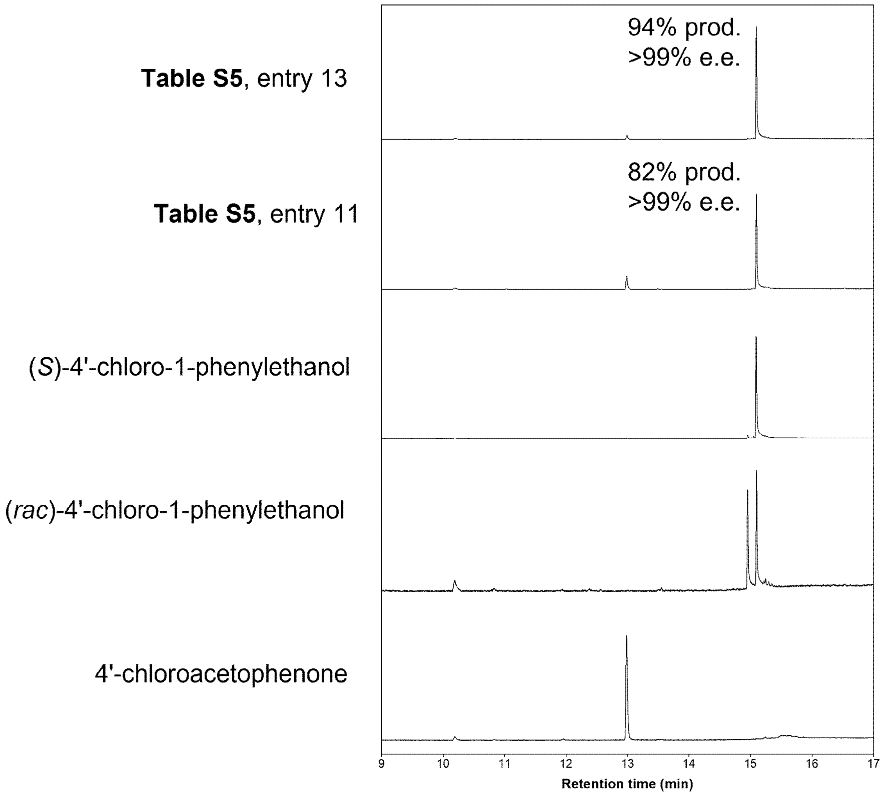

Figure S 30. Examples of chiral-GC results from reactions in **Table S 5** with the starting material and product standards. Standard for 4'-chloroacetophenone (available as 98% purity) and (*S*)-4'-chloro-1-phenylethanol (available as 95% purity) were purchased from Alfa Aesar and Sigma Aldrich respectively and diluted in ethyl acetate before injection. The racemic product standard was prepared by mixing 4'-chloroacetophenone with $NaBH_4$ in water. For each GC analysis, a 50 µL aliquot of the reaction filtrate or product standard was mixed with 800 µL EtOAc, then 600 µL of the separated EtOAc layer was removed and dried over Na_2SO_4.

S.2.17 Stability of NPs

As the NPs were prepared in batches to be used in the 4'-chloroacetophenone reduction, these were frozen after the NP synthesis (by flash freezing at -80 °C) and thawed slowly before use in reactions. The NPs were also “washed” using a size exclusion filter (10 kDa molecular weight cutoff, by centrifuging at 12,000 rpm for 10 mins) to remove any remaining metal salts. The UV-vis spectra after these stages are shown in **Figure S 31** and demonstrate the stability of the NPs to the freeze/thawing and to washing under centrifugal force; there is no noticeable change in the spectra after the freeze/thawing step, after washing there is some loss of absorbance which may have been due to some loss of NPs stuck to the filter but there is crucially no significant appearance of aggregated NPs.

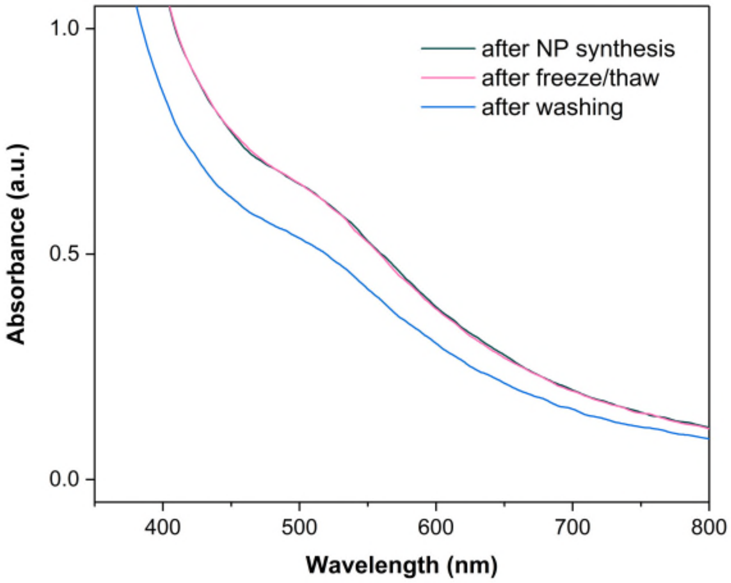

Figure S 31. UV-vis spectra of sample of *biohybrid NRase-Au@Pt NPs* recorded after the NP synthesis step, then after freezing at -80 °C and thawing and then after “washing” these NPs using a size exclusion filter.

S.3 References

[1] H. A. Reeve, L. Lauterbach, O. Lenz, K. A. Vincent, *ChemCatChem* **2015**, *7*, 3480-3487.
[2] W. Haiss, N. T. K. Thanh, J. Aveyard, D. G. Fernig, *Anal. Chem.* **2007**, *79*, 4215-4221.
[3] H. Jaegfeldt, *J. Electroanal. Chem. Interf. Electrochem.* **1981**, *128*, 355-370.
[4] Y. Shomura, M. Taketa, H. Nakashima, H. Tai, H. Nakagawa, Y. Ikeda, M. Ishii, Y. Igarashi, H. Nishihara, K.-S. Yoon, S. Ogo, S. Hirota, Y. Higuchi, *Science* **2017**, *357*, 928-932.
[5] L. Lauterbach, J. Liu, M. Horch, P. Hummel, A. Schwarze, M. Haumann, K. A. Vincent, O. Lenz, I. Zebger, *Eur. J. Inorg. Chem.* **2011**, *2011*, 1067-1079.
[6] J. S. Rowbotham, H. A. Reeve, K. A. Vincent, *ACS Catal.* **2021**, *11*, 2596-2604.
[7] I. V. Mironov, E. V. Makotchenko, *J. Solution Chem.* **2009**, *38*, 725-737.
[8] S. Wang, K. Qian, X. Bi, W. Huang, *J. Phys. Chem. C* **2009**, *113*, 6505-6510.
[9] A. J. Bard, L. R. Faulkner, *Electrochemical Methods: Fundamentals and Applications, 2nd Edition*, Wiley, **2001**.
[10] C. Greening, F. H. Ahmed, A. E. Mohamed, B. M. Lee, G. Pandey, A. C. Warden, C. Scott, J. G. Oakeshott, M. C. Taylor, C. J. Jackson, *Microbiol Mol Biol Rev* **2016**, *80*, 451-493.
[11] J. Drenth, G. Yang, C. E. Paul, M. W. Fraaije, *ACS Catal.* **2021**, *11*, 11561-11569.
[12] X. Liu, M. Atwater, J. Wang, Q. Huo, *Colloids Surf. B: Biointerfaces* **2007**, *58*, 3-7.
[13] V. Amendola, R. Pilot, M. Frasconi, O. M. Maragò, M. A. Iatì, *J. Phys.: Condens. Matter* **2017**, *29*, 203002.
[14] P.-A. Buffat, M. Flüeli, R. Spycher, P. Stadelmann, J.-P. Borel, *Faraday Discuss.* **1991**, *92*, 173-187.
[15] C. E. Paul, I. W. C. E. Arends, F. Hollmann, *ACS Catal.* **2014**, *4*, 788-797.
[16] A. Guarneri, A. H. Westphal, J. Leertouwer, J. Lunsonga, M. C. R. Franssen, D. J. Opperman, F. Hollmann, W. J. H. van Berkel, C. E. Paul, *ChemCatChem* **2020**, *12*, 1368-1375.
[17] S. Joseph Srinivasan, S. E. Cleary, M. A. Ramirez, H. A. Reeve, C. E. Paul, K. A. Vincent, *Angew. Chem. Int. Ed.* **2021**, *60*, 13824-13828.
[18] X. Zhao, S. E. Cleary, C. Zor, N. Grobert, H. A. Reeve, K. A. Vincent, *Chem. Sci.* **2021**, *12*, 8105-8114.

S.4 Author Contributions

Lucy B. F. Browne carried out the experimental work and formal analysis and wrote the majority of the original draft of this manuscript. Tim Sudmeier and Kylie A. Vincent advised on initial project ideas and experimental work. Maya A. Landis performed the TEM imaging. Christopher S. Allen performed the HR-STEM and EDX spectroscopy. All authors reviewed the manuscript. K. A. Vincent acquired funding to support this work.
